# Current advances in the development of microRNA-integrated tissue engineering strategies: a cornerstone of regenerative medicine

**DOI:** 10.3389/fbioe.2024.1484151

**Published:** 2024-10-16

**Authors:** Luis Germán Castañón-Cortés, Luis Alberto Bravo-Vázquez, Grecia Santoyo-Valencia, Sara Medina-Feria, Padmavati Sahare, Asim K. Duttaroy, Sujay Paul

**Affiliations:** ^1^ School of Engineering and Sciences, Tecnologico de Monterrey, Queretaro, Mexico; ^2^ School of Engineering and Sciences, Institute of Advanced Materials for Sustainable Manufacturing, Tecnologico de Monterrey, Queretaro, Mexico; ^3^ Department of Nutrition, Institute of Basic Medical Sciences, Faculty of Medicine, University of Oslo, Oslo, Norway

**Keywords:** microRNAs, tissue engineering, targeted delivery, regenerative medicine, tissue repair, gene regulation, gene therapy

## Abstract

Regenerative medicine is an innovative scientific field focused on repairing, replacing, or regenerating damaged tissues and organs to restore their normal functions. A central aspect of this research arena relies on the use of tissue-engineered scaffolds, which serve as structural supports that mimic the extracellular matrix, providing an environment that orchestrates cell growth and tissue formation. Remarkably, the therapeutic efficacy of these scaffolds can be improved by harnessing the properties of other molecules or compounds that have crucial roles in healing and regeneration pathways, such as phytochemicals, enzymes, transcription factors, and non-coding RNAs (ncRNAs). In particular, microRNAs (miRNAs) are a class of tiny (20–24 nt), highly conserved ncRNAs that play a critical role in the regulation of gene expression at the post-transcriptional level. Accordingly, miRNAs are involved in a myriad of biological processes, including cell differentiation, proliferation, and apoptosis, as well as tissue regeneration, angiogenesis, and osteogenesis. On this basis, over the past years, a number of research studies have demonstrated that miRNAs can be integrated into tissue-engineered scaffolds to create advanced therapeutic platforms that precisely modulate cellular behavior and offer a controlled and targeted release of miRNAs to optimize tissue repair and regeneration. Therefore, in this current review, we discuss the most recent advances in the development of miRNA-loaded tissue-engineered scaffolds and provide an overview of the future outlooks that should be aborded in this area of study in order to lay the groundwork for the clinical translation of these tissue engineering approaches.

## 1 Introduction

Regenerative medicine is one of the fastest growing fields in biotechnology, as it has been shown to have the potential of revolutionizing the strategies to treat a variety of diseases and even heal or ameliorate pathologies that previously lacked a practical treatment. Tissue engineering, which may be considered an area of regenerative medicine, seeks to generate specific neo-tissues or organs through the combination of different components, usually featuring biomaterials, cells and bioactive compounds. One common approach to this field is scaffold-guided tissue engineering, where a three-dimensional (3D) structure is used as a scaffold to guide cell growth into desired tissue configurations, with or without the aid. Some of the major variables for scaffold creation include its bulk material, 3D architecture, surface chemistry, mechanical properties and degradation dynamics. All of these characteristics allow for an endless range of designs, which should be chosen carefully according to the type of tissue that is being engineered and the mode of application; they may also be fabricated to be compatible with bioactive compound delivery strategies. It is also worth noting that not all tissue engineering developments aim to generate an implant: they may be employed for disease modeling, personalized diagnostics, or even advanced bioreactors (Chandra et al., 2020; Hutmacher et al., 2023; Uquillas et al., 2023).

Scaffolds by themselves may work effectively either as implants *in vivo* or as an environment for 3D cell culturing; however, the addition of biomolecules may yield even better results as it introduces chemical or biological stimuli to guide cell growth and interactions along with the physical stimuli provided by the scaffold (Chandra et al., 2020). Taking into account the fact that over 60% of gene expression in mammals is regulated by microRNAs (miRNAs) (Friedman et al., 2009), the potential of introducing these particular biological molecules to scaffolds becomes easy to elucidate. MiRNAs are non-coding RNA molecules with a length of around 22 nucleotides that can inhibit gene expression by binding to the 3′UTR region of messenger RNA (mRNA). This process is not specific to a single mRNA sequence, as miRNA can usually interfere with the expression of hundreds of genes in tissue-specific manners. MiRNAs have been found to have key roles in cellular processes of major relevance for tissue engineering, such as cell differentiation and proliferation, angiogenesis, innervation, scarring, inflammation and immune response; hence, multiple studies have looked into the therapeutic implementation of miRNA integrated tissue engineering strategies (Ribeiro et al., 2014; Beavers et al., 2015; Pu et al., 2019; Saliminejad et al., 2019). Nevertheless, a major challenge of the abovementioned strategies is the development of proper administration routes that ensure that it is localized in the proper tissue, which is important to maximize therapeutic efficiency and off-target effects prevention. Interestingly, loading miRNA onto scaffolds designed as grafts addresses such issues. Despite this, due to their negative charge, miRNAs struggle to get inside cells and exert their beneficial effects by themselves, and hence, it is necessary to develop approaches that could permeate miRNAs through the cell membrane. A wide array of options addressing this concern have been explored, like using components that work as carrier vehicles, including liposomes (LIPs), exosomes, nanoparticles (NPs), transfection complexes and functionalized metals. Additionally, miRNAs might also be coupled with molecules that enhance permeability, such as cholesterol, aptamers, and N-acetylgalactosamine (GalNAc), or even have chemical modifications like phosphorothioate. Viral vectors are also widely used for miRNA delivery. The implemented mechanism strongly influences how, when and for how long the small RNA biomolecule will be released onto the system and they may also be modified to allow for smart miRNA release dynamics or to create new interactions between the carrier and the scaffold (Saliminejad et al., 2019; Diener et al., 2022).

The development of innovative tissue engineering strategies has garnered scientific and economic interest as it may offer solutions for some of healthcare’s most serious concerns. The particular global challenges in which tissue engineering might have a significant impact are diverse; so far, these technologies have provided clinical breakthroughs for the reconstruction of the skin, mucosa, bone, articulations, bladder, ureter and even cardiac tissues and the spinal cord (Hoffman et al., 2019; Chandra et al., 2020; Paternoster and Vranckx, 2022). Undoubtedly, one of the exciting prospects of tissue engineering is organ replacement (Lewis et al., 2021). Although there is still a long way to go before whole organs can be fabricated, the development of tissue engineering technologies has and will continue to generate knowledge and practical applications for addressing multiple disorders. Additionally, this technology is accompanied by a high economic interest; for instance, just in the US, it was estimated that sales of tissue engineering products brought about $9 billion in 2017 (Kim et al., 2019).

In the following sections, recent updates on research featuring the use of tissue-engineered scaffolds in combination with miRNAs or anti-miRNAs will be summarized in order to gain insights into the current status and future potential of these strategies. The selection of scaffolds, miRNAs, delivery strategies and their effects on the analyzed models will be looked into within the contexts of different tissue groups, whether they are meant to aid in regeneration and functional recovery in models or provide a differentiation matrix for pluripotent cells.

## 2 MiRNA-integrated skeletal system tissue engineering strategies

The skeletal system is a vital component of the human body, providing structural support, protection for internal organs, and enabling movement. As well the skeletal system has a vital role as a framework that houses the bone marrow, where blood cells are produced (Frontera and Ochala, 2015; Watson and Adams, 2018; Mukund and Subramaniam, 2020). Despite its strength, the skeletal system is susceptible to various injuries and conditions, such as fractures, osteoporosis, and arthritis (Oryan et al., 2015; Heinlen and Humphrey, 2017; Adami and Saag, 2019). These issues can significantly impact mobility, quality of life and even lead to life-threatening complications if not properly managed (Beaudart et al., 2018). Remarkably, treating lesions of the skeletal system is challenging due to its complexity and the critical functions it performs. This necessitates a multidisciplinary approach involving healthcare professionals and advanced medical strategies such as interventional radiology (Barile et al., 2017; 2018).

Under such premise, tissue engineering scaffolds have arisen as promising alternatives for bone regeneration due to their particular characteristics, such as biocompatibility, non-toxicity, low cost, and non-carcinogenicity, coupled with outstanding osteoinductive and osteoconductive properties (Zeng et al., 2018; Alonzo et al., 2021). In addition, the use of bone tissue engineering scaffolds as gene therapy delivery systems offers promising prospects for enhancing bone healing and regeneration (Madry et al., 2020). Particularly, delivering miRNAs to the area of bone damage via scaffolds presents a promising therapeutic avenue for addressing bone defects and facilitating bone repair (Li et al., 2017; Guan et al., 2022).

In this context, Lei et al. (2019) developed a hydrogel for the directed and long-term co-delivery of miR-222 and aspirin (ASP). This hydrogel consisted of injectable thermoresponsive mesoporous silica NPs (MSN) embedded in a core-shell structured polymer composed of poly (ethylene glycol)-b-poly (lactic-co-glycolic acid)-b-poly (N-isopropylacrylamide) (PEG-PLGA-PNIPAM). The PLGA served as the core, while PEG/PNIPAM formed the shell of the hydrogel. Afterwards, researchers injected the miR-222/ASP MSNs hydrogel into a rat mandibular defect model and a synergistic effect of miR-222 and ASP was confirmed by noticing the generation of new bone tissue with neural-like structures in the area of the bone defect. Mechanistically, ASP was observed to promote bone formation, consistent with previous findings. Meanwhile, miR-222 was found to induce the differentiation of human bone mesenchymal stem cells (BMSCs) into neural-like cells via the Wnt/β-catenin/Nemo-like kinase signaling pathway. These findings suggest that this approach represents a promising avenue for engineering innervated bone tissue.

Later, an innovative method of miRNA delivery was developed using collagen-filled 3D-printed hybrid scaffolds. To attain this, hydroxypropyl cellulose-modified silver NPs were functionalized with miR-148b in order to transfect the miRNA into rat bone marrow-derived MSCs (rBMSCs). Subsequently, photoactivation at 405 nm allowed the release of miR-148b in the treated rBMSCs (Moncal et al., 2019). As a result, rBMSCs transfected with miR-148b showed early differentiation within collagen-filled 3D printed hybrid scaffolds, exhibiting notably higher levels of osteogenic markers in comparison to non-transfected rBMSCs. As well, scaffolds containing miR-148b-transfected rBMSCs significantly enhanced bone regeneration compared to scaffolds containing non-transfected rBMSCs. Furthermore, they facilitated nearly complete repair of critical-sized calvarial defects. These results offer evidence supporting the potential use of 3D-printed scaffolds containing miR-148b as a treatment option for future critical-sized bone defects.

Abazari et al. (2020) transducted human induced pluripotent stem cells (iPSCs) with miR-2861 and examined their osteogenic differentiation potential. Interestingly, this assessment was conducted while the cells were cultured on both an electrospun PLGA nanofibrous scaffold and a conventional culture plate. As a result, the miR-2861-transduced iPSCs exhibited significantly higher viability, mineralization, alkaline phosphatase (ALP) activity, calcium content, and expression of bone-related genes when compared to non-transduced iPSCs. Moreover, the outcomes of this study suggested that this enhancement is further augmented when miR-2861-transduced iPSCs are cultured on the PLGA nanofibrous scaffold, indicating a synergistic effect. Accordingly, this study highlights the significance of combined strategies using miRNAs and biomaterial scaffolds to regulate stem cell behavior and function, particularly in the context of tissue engineering.

Similarly, the outcomes of another investigation (Qi et al., 2021) indicated that incorporating miR-181a/b-1 did not exert any significant influence on the size and morphology of the nanofibers. Besides, it notably enhanced the biocompatibility of the nanofibers compared to those without miR-181a/b-1. Furthermore, the osteogenic differentiation capacity of human adipose tissue-derived MSCs (AT-MSCs) was upgraded when transduced with miR-181a/b-1 and this capacity was further synergistically increased when miR-181a/b-1 was incorporated into PLGA nanofibers. In fact, the AT-MSCs cultured on PLGA-miR nanofibers exhibited the highest expression levels of bone-related genes, i.e., runt-related transcription factor 2 (Runx2), collagen type-I (col-I), osteonectin (OSN) and osteocalcin (OSC). Overall, these results suggest that the combination of miR-181a/b-1 and PLGA nanofibrous scaffold holds promise for bone tissue engineering therapy.

Gan et al. (2021) reported that Chol-miR-26a (a cholesterol-modified miRNA) conjugated to an injectable PEG hydrogel showed selective targeting and significantly enhanced bone regeneration compared to the control groups. Particularly, the miRNA-conjugated gel was found to enhance ALP activity and promote calcium nodule deposition *in vitro* in MSCs. Moreover, in a rat model with critical skull defects, this therapeutic approach demonstrated the potential to repair these conditions. Therefore, injectable implantation with controlled release of osteogenic factors, such as Chol-c-miR-26a, shows promise for effectively treating large-scale bone defects.

Later, human umbilical cord-derived MSC-derived small extracellular vesicles (hUC-MSCs-sEVs) charged with miR-23a-3p were found to enhance calcium deposition and the formation of endothelial networks via the activation of the phosphate and tensin homologue (PTEN)/Protein kinase B (AKT) signaling pathway, inducing both osteogenic differentiation and angiogenesis. Furthermore, *in vivo* studies in skull defect model mice showed that a 3D-printed bioglass scaffold composed of gelatin methacryloyl (GelMA) and nanoclay loaded with hUC-MSCs-sEVs achieved vascularized bone regeneration through the slow release of the sEVs. In fact, the internalized sEVs promoted calcium accumulation and the development of an endothelial network, driving both osteogenic differentiation and angiogenesis by delivering miR-23a-3p. Thus, this innovative approach represents a possible therapeutic alternative for repairing critical-size bone defects (Hu et al., 2022).

Additionally, the combined administration of a miR-210 mimic and a miR-16 inhibitor using a collagen-nanohydroxyapatite scaffold system could present considerable promise for bone healing. As a matter of fact, this approach notably improved angiogenesis and osteogenesis in human MSCs, leading to increased calcium deposition within 10 days in two-dimensional (2D) culture and 14 days on scaffolds. In rats with calvarial defects, these scaffolds loaded with both miRNAs exhibited more than a twofold increase in bone volume, and the recruitment of blood vessels increased by 2.3 times compared to scaffolds without miRNA (Castaño et al., 2023).

Recently (in 2023), a 3D-printed scaffold made of tricalcium phosphate (TCP)/hydroxyapatite (HAp) in which hydroxyapatite NPs (HAp-NPs) modified with cationic functional molecules of 3-aminopropyltriethoxysilane (APTES) were utilized as carriers for miR-302a-3p, facilitated the delivery of this miRNA and promoted bone regeneration. Mechanistically, miR-302a-3p downregulated its target mRNA COUP-TFII (chicken ovalbumin upstream promoter-transcription factor II) and upregulated the expression of Runx2 mRNA, both of which are important for bone formation. *In vitro* tests using human mandibular-derived osteoblasts (HmOBs) showed efficient delivery of miR-302a-3p using this method. In a mouse model of calvarial defect, the modified scaffold induced significantly greater bone volume/total volume (BV/TV) and a greater number of filled spaces compared to controls. Besides, the histomorphometric analysis revealed that bone regeneration occurred more rapidly at the core of the HAp-NPs-APTES-miR scaffold compared to the control group. Consistently, this promising therapeutic approach should be further explored in order to develop a safe and efficient method to enhance the healing of critical-sized bone defects (Limlawan et al., 2023).

**FIGURE 1:**
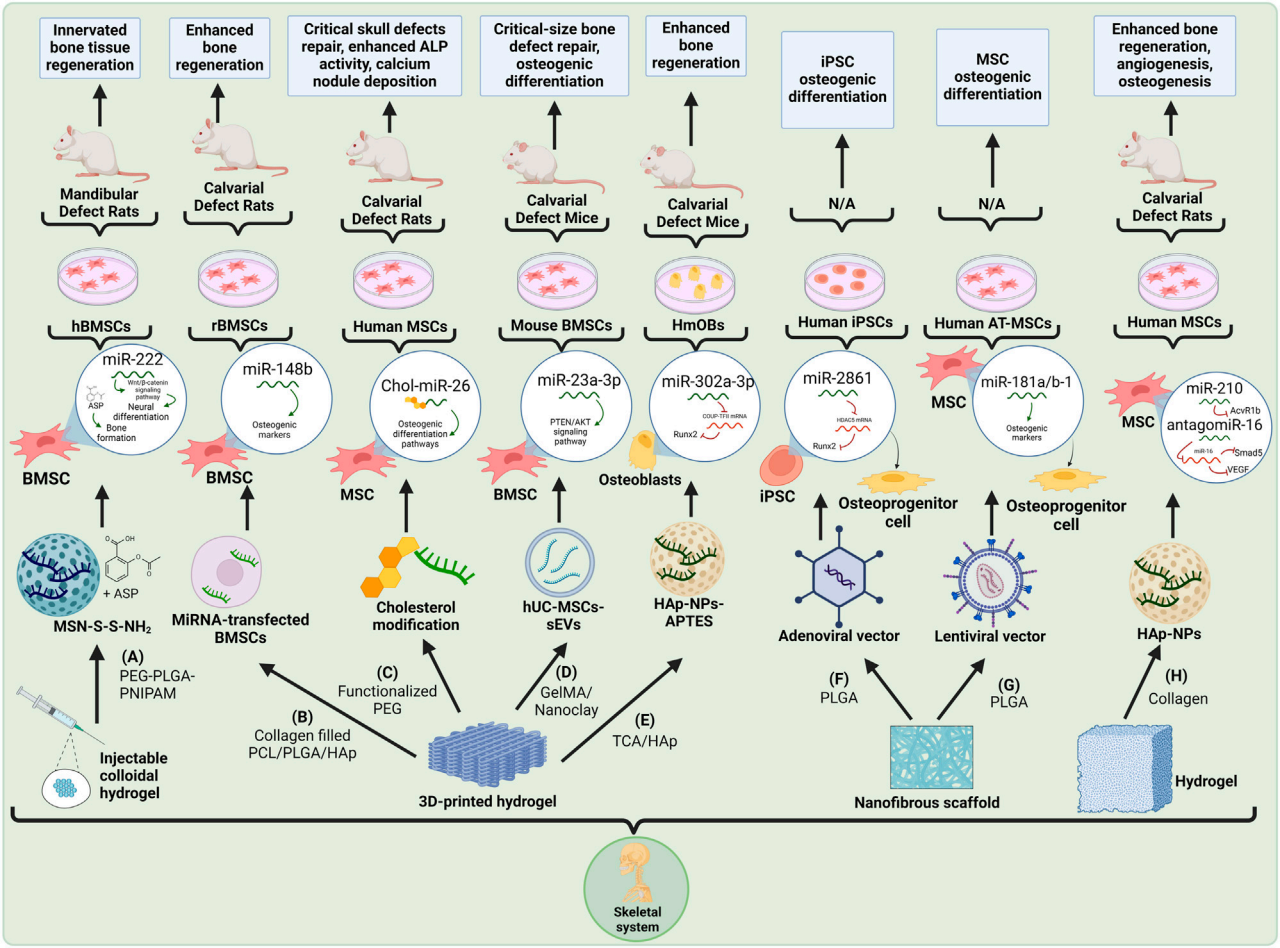
Schematic representation of recent miRNA-integrated skeletal system tissue engineering strategies: **(A)** A PEG-PLGA-PNIPAM injectable colloidal hydrogel with miR-222-loaded MSN and ASP for innervated bone tissue regeneration (Lei et al., 2019). **(B)** A collagen-filled PCL/PLGA/HAp 3D-printed hydrogel with miR-148b-transfected BMSCs for bone tissue regeneration (Moncal et al., 2019). **(C)** A functionalized PEG 3D-printed hydrogel with cholesterol-modified miR-26 for bone regeneration (Gan et al., 2021). **(D)** A GelMA/Nanoclay 3D-printed hydrogel with miR-23a-3p-loaded hUC-MSCs-sEVs for bone regeneration (Hu et al., 2022). **(E)** TCA/HAp 3D-printed hydrogel with miR-302a-3p-loaded HAp-NPs-APTES for bone regeneration (Limlawan et al., 2023). **(F)** PLGA nanofibrous scaffold containing adenovirus-transduced miR2861 iPSCs for iPSC osteogenic differentiation (Abazari et al., 2020). **(G)** PLGA nanofibrous scaffold containing lentivirus-transduced miR-181a/b-1 AT-MSCs for MSC osteogenic differentiation (Qi et al., 2021). **(H)** Collagen hydrogel scaffold with miR-210 and antagomir-16-loaded HAp-NPs for bone regeneration (Castaño et al., 2023). Created with Biorender.com.

Although the advances in the development of scaffold-mediated miRNA approaches centered on bone healing are progressive (Figure 1), it is worth emphasizing that the models that are currently used to study their effects possess certain limitations, such as the fact that they may not fully imitate the complex *in vivo* environment of the human system (Ehnert et al., 2020). Likewise, these models also display limited predictability and lack standardized protocols, making it difficult to compare the results obtained across different studies. In order to address these challenges, researchers may use more clinically relevant animal models to improve the translatability of their findings to humans (Anesi et al., 2020; Pfeiffenberger et al., 2021). Furthermore, the standardization of protocols to assess scaffold performance coupled with advanced imaging techniques to track bone regeneration can enhance the effectiveness and translational value of miRNA-loaded scaffolds in bone healing. Also, physiologists can improve their investigations via evaluating not only bone formation but also the quality of the regenerated bone tissue, as previously done in other assessments focused on bone regeneration (Veronesi et al., 2019; Dellavia et al., 2021; Płończak et al., 2023).

## 3 MiRNA-integrated nervous system tissue engineering strategies

Most of the recent research has focused on biomaterials-based tissue engineering strategies to promote the growth of nervous cells in the adult nervous system, which has a limited ability to heal itself (Madhusudanan et al., 2020; Cao et al., 2021; Doblado et al., 2021). Although tissue engineering might be used for a variety of complex neurological pathologies like neurodegenerative diseases (NDs), acquired brain injury (ABI) and traumatic brain injury (TBI), maximum research primarily concentrates on peripheral nerve injury (PNI) as a model for the peripheral nervous system (PNS) and, most commonly, spinal cord injury (SCI) as a model for the central nervous system (CNS). Ding et al. (2022) reported that in 2019, there were approximately 0.9 million new occurrences and 20.96 million preexisting cases of SCI. It is important to acknowledge that SCI, similar to other neurological illnesses, has enduring consequences that can greatly impact a patient’s quality of life. In the same year, SCI was responsible for a total of 6.2 million years lived with disability (YLDs).

Current traditional treatments are mainly capable of slowing down ongoing disease pathogenesis or preventing further damage (Madhusudanan et al., 2020); however, the studies discussed in this section aim to contribute to the regeneration of nervous tissue, ideally leading to functional recovery. In this endeavor, miRNAs have been valuable biomolecules as they regulate important biological processes that aid in functional recovery, such as neural stem cell proliferation and differentiation into neuron or glial lineages, neurite extension and regeneration, myelin formation and inhibition of inflammatory responses and glial scar formation (Nguyen et al., 2015; Tang and Sun, 2020; Silvestro and Mazzon, 2022). Despite its efficacy, delivery of miRNA is quite challenging for the CNS since it is isolated by the blood-brain barrier; however, embedding these molecules into scaffolds allows them to be delivered *in situ*. Furthermore, scaffolding provides mechanical cues for cell growth, which is important as having the proper cell architecture aids in functional recovery. Therefore, combining miRNA delivery with scaffold implantation is a promising strategy for nervous tissue engineering, even more so considering that conditions like SCI will often require surgical management regardless of the introduction of regenerative medicine approaches (Wang et al., 2021). While some of the current studies focus on the activation of endogenous cells for regeneration, cell transplantation has also been shown to yield positive effects; nevertheless, a major challenge of cell therapy approaches in nervous tissue engineering is sourcing cells, as the grafted cells should have a human, non-malignant origin and, ideally, it should be an autograft. Nervous cells with these characteristics are scarce and hard to obtain ethically, but scaffold and miRNA technologies featured in this section have been used to tackle this as they can support the differentiation of stem cells, like iPSCs and MSCs, into neuron and glia progenitors like neural stem cells (NSCs), or the selective differentiation of the latter into specific cell lineages like neurons or glial cells.

The use of fiber-hydrogel scaffolds for miRNA delivery in SCI models has been growing over the last couple of years. In such systems, aligned fibers are used to biomimic the architecture of the microenvironment in which neurons and glia naturally grow and direct axon growth, while the hydrogel component supports diffusion-mediated delivery of pharmaceutical agents, such as miRNAs complexed with a carrier. For instance, Milbreta et al. (2019) used electrospun poly (caprolactone-co-ethyl ethylene phosphate) (PCLEEP) fibers embedded in a col-I hydrogel to create a hybrid scaffold which supported neurotrophin-3 (NT-3) transport along with TransIT-TKO (TKO)-mediated miR-219 and miR-338 delivery in SCI models. Such miRNAs were selected to enhance remyelination, as they have been shown to promote the differentiation of endogenous oligodendrocyte progenitor cells (OPCs) into oligodendrocytes (OLs) by suppressing OPC proliferation-related gene targets. NT-3 was included as it is a neurotrophic factor that aids in neuron survival. In this research, primary rat OPCs purified from postnatal day 0 to day 2 neonatal rat cortices were transfected into the hybrid scaffolds, showing enhanced OPC differentiation and maturation, increased myelin sheath length and more complete-looking myelin structures in miR-219/miR-338 scaffolds in contrast to negative scrambled miRNA (Neg miRNA) scaffolds. Moreover, it was found that over the first hour following transfection, around 10% of loaded miRNAs and NT-3 were released; for the next 20 days, about 65% of miRNAs and 70% of NT-3 were steadily released and, finally, the release profiles plateaued at day 60. Adult female Sprague-Dawley rats were subject to a C5-C6 hemisection followed by scaffold implantation. Models implanted with miR-219/miR-338 scaffolds preserved the number of oligodendroglial lineage cells around the injury site, promoted OPC differentiation and enhanced myelin formation after SCI when compared to the Neg miRNA group; additionally, it was found that miR-219/miR-338 delivery did not affect neurite outgrowth or glial scarring. The results seem to point out that this is a promising scaffold for SCI and perhaps for demyelinating diseases, too (Milbreta et al., 2019).

Zhang et al. (2019) employed a fiber-hydrogel scaffold as well. Electrospun polycaprolactone (PCL) was selected as the fiber due to its favorable mechanical properties, biocompatibility and biodegradability; additionally, the fibers were coated with poly-3,4-dihydroxy-l-phenylalanine (poly-DOPA) to allow for adsorption of laminin, which enhanced neuron attachment, and the TKO-complexed miRNAs. Four different miRNAs and their combinations were tested: miR-21, which targets a gene that antagonizes fibroblast growth factor (FGF) signaling; miR-132, which targets genes related to cytoskeletal regulation; miR-222, which targets an inhibitor of central axon growth; and miR-431, which enhances neurogenesis, axon growth and motor neuron axon outgrowth. *In vitro* studies were performed with rat cortical neurons extracted from embryonic day 14 and day 1 pups, as well as adult Sprague-Dawley rats dorsal root ganglion (DRG) neurons; in these, neurite length was assessed for each treatment and neuron group by fluorescence microscopy. It was observed that patterns were similar regardless of the neuron group and the top four miR treatments were determined: miR-21, miR-132, miR-222/miR-431, and miR-132/miR-222/miR-431. Quantitative polymerase chain reaction (qPCR) showed that the mRNA targets for each of the miR involved in the top four treatments: Sprouty2 for miR-21, PTEN for miR-222, Kremen1 for miR-431 and p120RasGAP (Rasa1) for miR-132 were decreased in comparison to Neg miRNA-treated groups. It was also observed that treatments enhanced the formation of growth cones by immunostaining neurons of each of the top four treatments. Finally, for *in vivo* analyses, scaffolds were fabricated with the same principles but slight variations: the fibers were made out of electrospun PCLEEP instead of PCL, embedded in a col-I matrix and NT-3 was added along the top miRNA cocktails featuring 1, 2 and 3 different miRNAs. These scaffolds were tested on female Sprague-Dawley rats with SCI induced by T9-T10 hemisection and after 2 weeks, neurofilament growth parallel to the spinal cord was enhanced on scaffolds containing miRNAs, as opposed to scaffolds containing NT-3 only. No obvious glial scarring was observed, showing that these scaffolds are a highly valuable approach for treating SCI, with the combination of miR-132/miR-222/miR-431 showing the highest neurite density *in vivo* (Zhang et al., 2019).

The combination of iPSC, miRNA and scaffold technologies has been explored as a source of human neurons and glia. Nazari et al. (2020) transduced human fibroblast iPSCs with a miR-338 lentiviral vector, which is known to play a crucial role in OL differentiation by targeting transcription factors Sox6, Hes5, and ZFP238, in order to assess the effect of scaffolds as matrices for induction of OL differentiation. The scaffolds they tested were made of fibrin (FB) hydrogel, which is a polymer that naturally occurs during blood clotting and is known to promote cell adhesion and proliferation, which can be used for extrusion printing and direct injection. The differentiation on FB hydrogel was contrasted with tissue culture plates, treating miR-338 transduced iPSCs with induction media in both conditions over 18 days. 3-(4,5-dimethylthiazol-2-yl)-2,5-diphenyltetrazolium bromide (MTT) assay showed an increase in viability when using FB hydrogel, while the morphology of OLs was assessed through scanning electron microscopy (SEM). Most importantly, immunofluorescence assays and qPCR analysis showed that the expression of OL-associated markers was higher when differentiating on the scaffolds. This study shows that combining miRNA and scaffold technologies could be an effective way of sourcing nervous system-relevant cell lines and might even support differentiation *in situ*.

Similarly, Jedari et al. (2020) investigate the effects of using scaffolds and miRNAs as differentiation environments for applications in neuroscience. MiR-7 upregulation has been observed in brain and cortical neural progenitor cells; therefore, it was selected for lentiviral transduction of trabecular meshwork MSCs (TMMSCs) in order to differentiate them into glial and neural progenitor cells. In this case, nanofibrous scaffolds composed of electrospun poly-L-lactic acid (PLLA) and PCL were employed to grow and differentiate the TMMSCs. Immunostaining and qPCR were used to assess neural marker expression, particularly nestin, glial fibrillary acidic protein (GFAP) and microtubule-associated protein-2 (MAP-2). This study showed that miR-7 overexpression could lead to neural differentiation and the PLLA/PCL scaffold can further improve differentiation yields.

Following up on the work they published in 2019 (Zhang et al., 2019), Zhang et al. (2021) revisited the PCL fiber and col-I hydrogel scaffold with miR-132/miR-222/miR-431; however, they used glial cell-derived neurotrophic factor (GDNF) instead of NT-3 and looked into neurological and functional recovery of the rat SCI models over 12 weeks instead of two. Fluorescence microscopy revealed that this approach significantly regenerated mature axons as well as serotonergic axons at the rostral region and sensory axons at the caudal region. Furthermore, the addition of methylprednisolone (a glucocorticoid steroid previously reported to aid in neurological recovery) to the miRNA-delivering scaffold allowed for an enhanced myelination index, along with functional recovery, as shown by Basso, Beattie and Bresnahan (BBB) locomotor scale method and paw withdrawal tests. RNA sequencing showed that the treatment downregulated pro-inflammatory genes, while extracellular matrix (ECM) deposition genes were upregulated; additionally, a transcriptome profile for the rat SCI models was established. Through this research, the results from the previous studies were improved upon and more valuable data regarding the therapeutic potential of these scaffolds was obtained.

In 2022, an interesting study regarding the effect of nanofibrous scaffolds on the differentiation of NSCs into neurons was conducted (Li et al., 2022). A silk fibroin (SF) nanofibrous scaffold was created using supercritical CO_2_ technology. As opposed to previous studies which used lentiviral vectors to induce miRNA with the objective of aiding in cell differentiation, this study used chitosan NPs (CS NPs) loaded with miR-222, which was used by Zhang et al. (2019) and Zhang et al. (2021) too, as discussed earlier in this section. CS was chosen as a carrier due to its biocompatibility and previous reports on enhancing drug delivery *in vivo*. The nanofibrous SF scaffolds were also coated with poly-dopamine (PDA), as this allows to anchor of the CS NPs onto them. Mouse neuroepithelial cells NE-4C were seeded onto the SF scaffold with CS NP@miR-222 and cultured over a week with a differentiation medium. Using nestin as a marker for undifferentiated cells, GFAP as a marker for astrocytes, Tuj-1 as a marker for neurons and β-actin as a calibrator, Western blot and qPCR assays showed that the cells treated on an SF scaffold with CS NP@miR-222 showed more neural differentiation than the rest of the analyzed groups.

Liu et al. (2022) also conducted experiments on SCI models using one of the miRNAs that Zhang et al. (2019) had used: miR-21. For this purpose, Exos were engineered to be used as miR-21 carriers and they were loaded onto col-I hydrogel scaffolds. A fusion protein was designed to have an extracellular collagen-binding domain (CBD) and a transmembrane lysosome-associated membrane glycoprotein 2b (Lamp2b) domain; then it was transduced into HEK293T cells by lentiviral vectors to produce CBD-LP-Exos. In order to create CBD-LP-Exos containing miR-21, or CBD-LP-miR-21-Exos, transient overexpression of miR-21 was induced by a lentiviral vector and the Exos was extracted. Col-I hydrogel scaffolds were fabricated and then soaked in a solution containing CBD-LP-miR-21-Exos to obtain CBD-LP-miR-21-EXO-Col. Female Sprague-Dawley rats with a T10 complete transection were used as *in vivo* models of SCI. The scaffolds were implanted on the injury sites and were observed over 8 weeks. BBB scores showed that rats receiving CBD-LP-miR-21-EXO-Col had a significantly better functional recovery, while histological analyses revealed smaller cavities at SCI sites, lower expression or markers of glial scarring and higher neuronal markers. Additionally, the degradation dynamics of the scaffold were assessed, finding that it degraded 8 weeks post-implantation, which is desirable as it is supposed to be replaced by endogenous tissue.

The studies so far have focused on SCI models, but Wang et al. (2023) described the development of a tissue engineering strategy featuring miRNA delivery for PNI. This endeavor is often tackled by the use of a nerve guidance conduit (NGC), which acts as a scaffold and directs the growth of peripheral nerves. NGCs require a good mechanical strength, as it is directly subjected to stretching and extrusion of muscle tissue, and this research team proposes the use of a hydrogel composed of SF and gelatin-tyramine (GT), which has favorable mechanical properties and biocompatibility. MiR-29a was chosen as it has been observed to promote axonal elongation by regulating the Phosphatidyl inositol 3′-OH kinase (PI3K)/Akt/Mammalian target of rapamycin (mTOR) pathway, and it was coupled with a metal-organic framework carrier, ZIF-8, so that it can be delivered into cells. Rat Schwann cells RSC96 were seeded in the SF/GT/miR-29a@ZIF-8 NGC, showing good growth and proliferation. Likewise, rat adrenal medulla pheochromocytoma cells PC12 were seeded on these NGC to assess differentiation markers by qPCR and Western blot analyses, and it was found that, while SF/GT/ZIF-8 could enhance neural differentiation, probably due to the release of Zn^2+^ ions, SF/GT/miR-29a@ZIF-8 had the highest expression of neural markers and axon elongation.

Most recently, miR-29a has also been studied for its use in SCI. Wan et al. (2023) immobilized miR-29a onto gold NPs (AuNPs), which had been coated with O-(2-Mercaptoethyl)-O′-methyl PEG (PEG-SH) in order to interact with the miRNA. The PEG-SH-AuNPs@miR-29a were then loaded onto a self-assembling peptide nanofiber scaffold (SAPNS). The effect on neural differentiation was assessed *in vitro* with rat NSC using immunofluorescent staining, showing that NSCs differentiated into nestin (+) glial cells, Map-2 (+) neuron-like cells and β3-Tubulin (+) neuron-like cells, all with decent cell activity as indicated by Hoechst 33342 staining. Visual analysis revealed that cells seeded on PEG-SH-AuNPs-SAPNS@miR-29a had a greater length, density and number of neurites than the rest of the groups. Male Sprague Dawley rats with T10 thoracic cord injury were used as *in vivo* models, which also had enhanced neurite development and BBB score when treated with PEG-SH-AuNPs-SAPNS@miR-29a. This showed that this treatment led to neurite regeneration and functional recovery in the tested SCI models.

**FIGURE 2:**
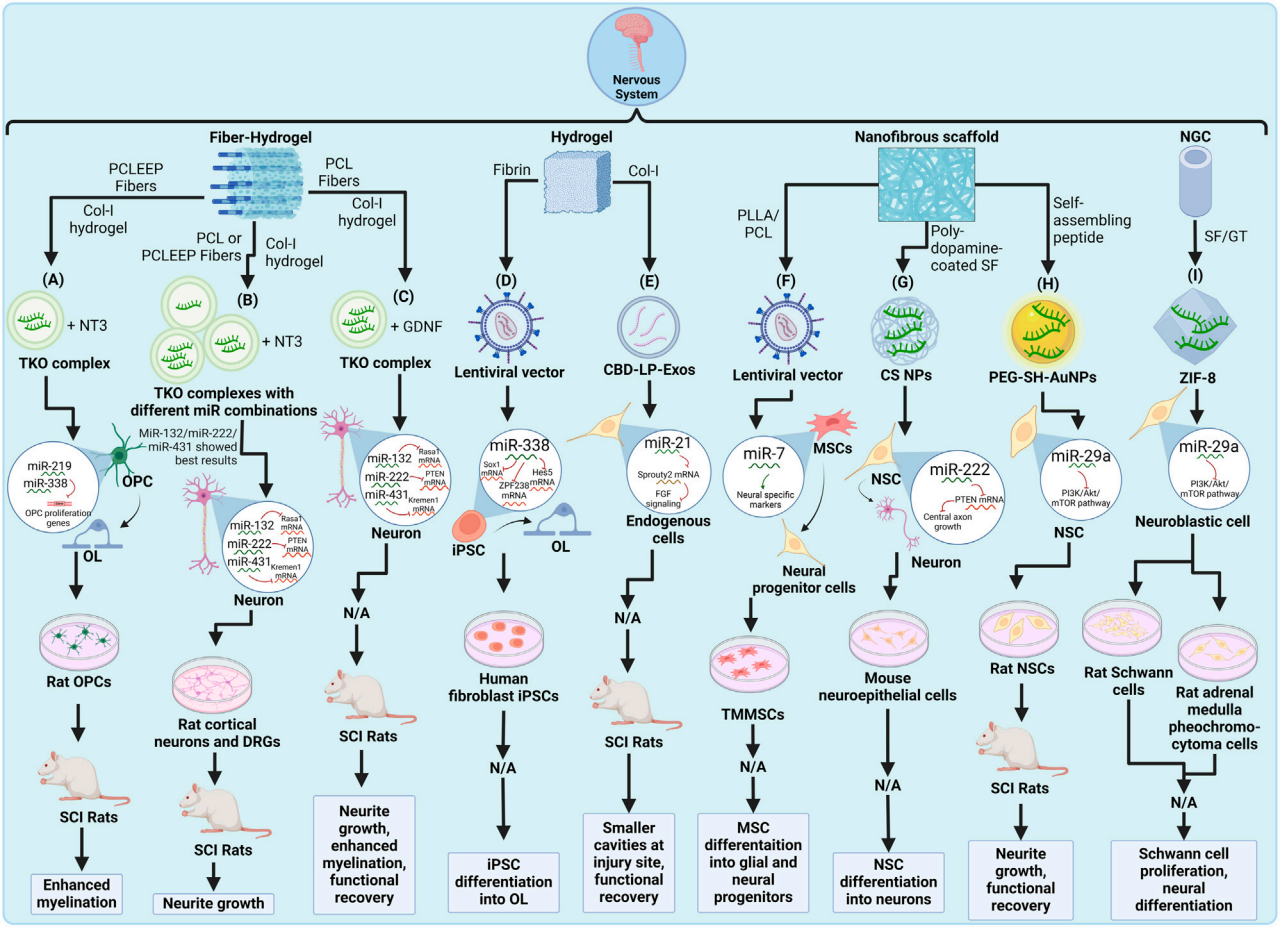
Schematic representation of recent miRNA-integrated nervous system tissue engineering strategies: **(A)** A PCLEEP/Col-1 fiber-hydrogel scaffold with miR-219 and miR-338 complexed to TKO and NT-3 for SCI (Milbreta et al., 2019). **(B)** PCL/Col-1 fiber-hydrogel scaffolds with combinations of miR-21, miR-132, miR-222 and miR-431 complexed to TKO and NT-3 for spinal cord injury (Zhang et al., 2019). **(C)** PCL/Col-1 fiber-hydrogel scaffold with miR-132, miR-222 and miR-431 complexed to TKO and GDNF for SCI (Zhang et al., 2021). **(D)** A fibrin hydrogel scaffold containing lentivirus-transduced miR-338 iPSCs for iPSC differentiation into glial and neural progenitors (Nazari et al., 2020). **(E)** Col-1 hydrogel scaffold with miR-21-loaded CBD-LP-Exos for SCI (Liu et al., 2022). **(F)** A PLLA/PCL nanofibrous scaffold containing lentivirus-transduced miR-7 TMMSCs for MSC differentiation into oligodendrocytes (OL) (Jedari et al., 2020). **(G)** A poly-dopamine-coated SF nanofibrous scaffold with miR-222-loaded CS NPs for NSC differentiation into neurons (Li et al., 2022). **(H)** A self-assembling peptide nanofibrous scaffold with miR-29a-loaded PEG-SH-AuNPs for SCI (Wan et al., 2023). **(I)** A SF/GT nerve guidance conduit (NGC) with miR-29a-loaded ZIF-8 for peripheral nerve injury (PNI) (Wang et al., 2023). Created with Biorender.com.

This compilation of studies showed the recent developments in strategies combining miRNA therapeutics and tissue engineering (Figure 2). While the results for most experiments are promising, it is important to take into account that regenerative medicine approaches for neurological pathologies such as SCI are still in the early stages of development since clinical trial data is scarce and reproducibility remains challenging (Zipser et al., 2022). In the case of scaffolds incorporating miRNA, there’s no information available regarding human trials and a couple of the discussed studies did not even feature animal trials. It is also important to note that while all of the experiments showed better results when using miRNA-loaded scaffolds than when leaving the injury untreated or not delivering miRNA, a complete functional recovery has not been achieved by any treatment. Nevertheless, even partial motor or sensory function restoration may be significant in improving a patient’s quality of life (Goulet et al., 2019; Huang et al., 2020); therefore, these studies are extremely valuable for the development of strategies with clinical application and might elucidate a framework for tending even more complex CNS and PNS diseases.

## 4 MiRNA-integrated cardiovascular system tissue engineering strategies

Tremendous strides have been made in the field of tissue engineering, particularly in the context of addressing cardiovascular diseases (CVD), which remains a leading cause of disease burden worldwide (Roth et al., 2020). The integration of scaffold-mediated miRNA delivery represents a promising frontier in this endeavor. Cardiac tissue engineering (CTE) scaffolds aim to mimic the fibrillar structure of the ECM in order to replicate the native 3D environment, facilitating the regeneration of damaged or diseased cardiovascular tissues (Gil et al., 2022). CVD consists of a wide range of conditions, including myocardial infarction (MI), heart failure (HF), and ischemic heart disease, all of which pose significant challenges to public health globally (Peer et al., 2021; Roger, 2021). With the prevalence of these diseases on the rise, there is a pressing need for effective therapeutic strategies that can mitigate their impact. Scaffold-mediated miRNA delivery holds immense potential in this regard, offering targeted and personalized treatment options for managing cardiovascular ailments (Sharma et al., 2021). Central to this approach are the key cellular players involved in cardiovascular regeneration, including but not limited to cardiomyocytes (CM), endothelial cells (ECs), smooth muscle cells (SMCs), and cardiac fibroblasts. These cells play vital roles in maintaining cardiac function and responding to injury or disease.

From the nucleic acid therapies, regulation by miRNAs is one of the most likely to be used for vascular regeneration since they play critical roles in tissue regeneration by regulating gene expression and modulating cellular processes (Wen et al., 2019; Muniyandi et al., 2021). Incorporating miRNAs into scaffold-based delivery systems offers several advantages, including enhanced stability, targeted delivery, and sustained release kinetics. This way, researchers can accurately control the geographical and temporal distribution of miRNAs, maximizing their therapeutic potential and limiting off-target effects (Sharma et al., 2021). However, despite its promise, CTE and drug delivery in this system present unique challenges, including achieving proper scaffold integration. For example, difficulties for small-caliber vascular grafts include the risk of thrombosis, intimal hyperplasia, restenosis, or calcification (Wen et al., 2019; 2020; Li et al., 2021) while for the miRNAs, ensuring sustained miRNA delivery to target cells, retention, stability, and degradation are some of the most featured challenges (Li et al., 2021) Overcoming these obstacles requires innovative strategies that address the specific demands of CTE and drug delivery.

One of the many uses for scaffolds in regulation by miRNA therapy for the clinical management of vascular diseases, is their application in small-diameter vascular regeneration. Wen et al. (2019) prepared a poly (ethylene glycol)-b-poly (l-lactide-co-ε-caprolactone) (PELCL) electrospun membrane with the intention of controlling the phenotype and proliferation of SMCs, which is crucial to dealing with thrombosis and restenosis. PELCL was selected for its biocompatibility and appropriate mechanical characteristics. For this study, three Val-Ala-Pro-Gly (VAPG) peptide-modified trimethyl chitosan-g-PEG (TMC-g-PEG-VAPG) polymers with different chitosan molecular weights were created in order to distribute miR-145 to SMCs in the PELCL electrospun membranes. MiR-145 was used since it can control SMCs to maintain a normal contractile phenotype and prevent intimal hyperplasia and excessive proliferation. For targeted delivery of this miRNA, TMC-g-PEG-VAPG (TPV) was developed, enhancing cellular uptake in SMCs. The TPV/miR-145 complexes showed minimal cytotoxicity, and TPV with a higher molecular weight (50 kDa) could significantly boost cellular absorption in SMCs. Furthermore, within 72 h, the electrospun PELCL membranes were able to regulate SMCs at the gene and protein levels. Additionally, the electrospun membrane’s released miR-145 demonstrated *in vitro* bioactivity by altering the contractile phenotype of SMCs for a minimum of 56 days.

Later, another attempt to prevent the problems related to small-caliber vascular grafts was made by Wen et al. (2020) by developing a trilayered bioactive tissue-engineered vascular grafts (TEVGs) encapsulating both miR-126, which can quicken the production of fibroblast growth factor and vascular endothelial growth factor, and miR-145, that regulates the phenotype of contractile SMC in the structural inner and middle fibrous layers, correspondingly. In order to ensure local delivery of the encapsulated miRNAs and to enhance the attachment and proliferation of ECs, the arginine-glutamic acid-aspartic acid-valine (REDV) peptide was used. To resemble the natural ECM, PELCL was used, since it is an effective TEVG due to its flexibility, biodegradability, and biocompatibility. The electrospun membrane was created with the inner and middle layers of PELCL, with the inner including REDV, while the outer layer was composed of PCL. This allowed to release of miR-126 quickly through a REDV peptide-modified TMC-g-PEG (TPR) complex in the inner layer and miR-145 slowly through TPV in the middle layer. To assess the impact of released miRNAs on cell proliferation, human umbilical vein ECs (HUVECs) were cultured *in vitro* for 9 days on tri-layered membranes. It had notable biological benefits in terms of vascular ECs’ increased proliferation, intracellular nitric oxide generation, SMC phenotypic modification, and calcium deposition inhibition. The trilayered electrospun grafts were implanted in rat abdominal arteries for 4 and 12 weeks in order to conduct an *in vivo* evaluation. The dual-miRNA-loading trilayered electrospun graft appeared to have a beneficial impact on contractile SMC regeneration, endothelization, and proper ECM production, according to histological and immunofluorescent investigations. In addition, the trilayered vascular graft’s local bioactivity of miR-126 and miR-145 may help macrophages (MCs) change into the anti-inflammatory M2 phenotype, which would control inflammation and reduce calcification.

On the note of innovative strategies to address CVD, Li et al. (2021) created a drug delivery system to mitigate the inflammatory response after MI, restore compromised heart function, and enhance angiogenesis to preserve and restore the blood flow of the surviving ischemic myocardium. The approach involves miR-21-5p, a proangiogenic treatment delivered by amino (-NH2) and trimethylamine functionalized MSNs (MSN-NH2-TMA), an anti-inflammatory nanomaterial that, when phagocytosed by MCs, blocks the proinflammatory (M1) phenotypes. These NPs are encapsulated in an injectable hydrogel matrix (Gel@MSN/miR-21-5p) that precisely releases the MSN/miR-21-5p complexes solely in the acidic infarct area. To assess the *in vitro* bioactivity of MSN/miR-21-5p complexes released from Gel@MSN/miR-21-5p were cultured with ECs and then cocultured with CM that had experienced ischemia or hypoxia. Primary peritoneal MCs obtained from female C57BL6J mice were also tested with Gel@MSN/miR-21-5p to assess immunomodulatory effects *in vitro*. According to the research, proangiogenic factors were enhanced and proinflammatory cytokines were decreased by the MSN/miR-21-5p complexes. Under ischemia and hypoxic conditions, the elevated proangiogenic factors from ECs could successfully prevent CM from apoptosis. *In vivo*, the released MSN complexes in a pig MI model substantially suppressed inflammation by polarizing M1 MCs within the infarcted myocardium. Moreover, additional miR-21-5p distribution to ECs by MSNs aided in the rescue of at-risk CM and encouraged local neovascularization. The potential of this method for treating MI was demonstrated by the considerable reduction in infarct size that resulted from the combined anti-inflammatory and proangiogenic effects.

Sustained delivery of miRNA for therapeutic purposes continues to be challenging. In order to overcome delivery system limitations and improve reprogramming efficiency, CTE involves deciphering cellular and molecular principles pertaining to heart regeneration and devising an effective reprogramming approach. For this purpose, Muniyandi et al. (2021) used miRNAs as reprogramming factors for transdifferentiation and the creation of functional cardiac patches (CP) from somatic cells. A functional scaffold was constructed with PLLA, a hydrophobic, biocompatible, biodegradable linear polymer with good spinnability that is approved by the United States Food and Drug Administration (FDA). This was achieved through electrospinning, which creates nanofibers with adjustable characteristics. These electrospun nanofibers present a high surface area with interconnecting pores, which helps with cell adhesion and retention, possessing superior mechanical properties with outstanding biocompatibility. To attain sustained delivery of miR-1, an essential cardiogenesis regulator that promotes differentiation in the embryoid body, and miR-133a, a cardioprotective molecule that targets epidermal growth factor receptors to aid in differentiation, polyethyleneimine (PEI) was used (PEI-miRNA complexes). Due to PEI being positively charged, it is commonly used for transfection, acting as an efficient proton pump that promotes endosomal enlargement and rupture, as well as increases RNA release from the endosomal complex inside the cells. Intriguingly, PEI-miRNA complexes were immobilized on PLLA scaffolds with smooth and porous structures, resulting in a loading efficiency of ∼96% for the transfected adult human cardiac fibroblasts (AHCF) cultured on fibronectin-coated PLLA surfaces regardless of their surface topology. These scaffolds significantly increased the reprogramming efficiency and activated early transcription factors when AHCF were reprogrammed to induced CM (iCM)-like cells. This time, compared to the smooth PLLA scaffolds, the reprogrammed cells on the porous scaffolds had a higher intensity. Moreover, PEI-miRNA polyplexes showed a biphasic release pattern from the scaffolds in the *in vitro* release experiment. Due to their combinatorial effect, including the topographic inputs of electrospun fibers and dual miRNAs’ ability to accurately control the fate of cardiac fibroblast cells, these dual miRNA scaffold systems turned out to be an effective formulation (Muniyandi et al., 2021).

Another effort to overcome delivery obstacles was made by Van der Ven et al. (2021) by developing a promising polymer-NP (PNP) based hydrogel, which uses PEG-block-Poly-(lactic acid) (PEG-*b*-PLA) as the polymer. This polymer has the ability to flow under shear strain and is capable of reforming when the external stress is released, indicating its shear-thinning and self-healing properties, which result from the polymer’s reversible non-covalent interactions with the gel’s NPs. In their study, they fused miRNA-mimics to AuNP that were functionalized with PEG to lessen degradation and increase biostability. Subsequently, these AuNP-miRNAs were loaded into a biocompatible injectable hydrogel that is based on the PNP gel platform in order to enhance the administration of the miRNA in a localized, sustained, and less invasive manner. Moreover, endosomal escape was stimulated intracellularly by adding influenza hemagglutinin 1 peptide, which destabilizes the endosomal membrane. To assess the controlled release, AuNPs were functionalized with the cel-miR-67, which has no biological effect on human cells, and loaded into the PNP hydrogel, observing a sustained linear release of the loaded AuNPs over a period of 5 days, up to 20%. Subsequently, the authors demonstrated the *in vitro* mRNA targeting activity by combining the AuNPs with miR-214 (known to raise the calcification of human aortic valve interstitial cells) and incubated with HEK293 cells transfected with a miR-214 target luciferase reporter, resulting in a significant 33% reduction of the Luciferase signal. Furthermore, it was observed that AuNP-miRs maintain their functionality in a more intricate tissue model by monitoring labeled AuNP-miR-67 in a 3D bioprinted human model of calcific aortic valve disease (CAVD); this showed that the cell absorbed AuNP-miR-67 through lysosomes or endocytosis. Additionally, AuNP-miR-214 increased the expression of ALP, which is associated with early calcification in CAVD. Finally, the distribution of PNP-AuNP-miR-67 throughout the body was assessed after injecting it under the skin of C57BL/6 mice. After 11 days of injection, it was observed that the liver and kidneys were mostly responsible for the clearance of AuNP-miR-67, and the fluorescence levels in these samples returned to the same level as the control group (Van Der Ven et al., 2021).

Over the past 30 years, the development of synthetic CPs has been the primary focus of CTE research, with a special focus on extrusion-based 3D printing since it is an affordable and simple technique that allows the use of multiple biomaterials. However, these 3D-bioprinted CPs have comparatively low cell viability due to the shear force induced on them when the bioink is pushed through a needle. Therefore, it was theorized by Bar et al. (2023) that the viability within the CP would be increased by the cell survival factor miR-199a-3p. Studies have shown that this human miRNA is involved in the activation of the transcriptional cofactor YAP, a well-established signaling pathway that controls the proliferation of CM and has been shown to promote cardiac regeneration in adult mice models with MI. EVs derived from activated human monocytic THP-1 cells were selected to deliver this miRNA based on the assumption that these cells would communicate with other cardiovascular cells since Monocyte-derived MCs (MΦ) use them as a signaling pathway to interact with neighboring cells and take part in cardiac repair after MI. EVs also participate in CM survival, which ceases to proliferate after heart damage, decrease inflammation, and reduce oxidative stress *in vitro* and *in vivo*. In addition, their usual cargos are mRNA and miRNA. This way, a proposed M1-like MΦ derived-EV system was designed to sustainably deliver miR-199a-3p. To validate that the MΦ-derived EVs were internalized by their target cells, engineered EVs were evaluated in neonatal rat CM (NRCM) monolayers, demonstrating their applicability. The inclusion of these EVs in bioink, consisting of alginate-RGD, gelatin, and NRCM, resulted in CPs that tripled their cell vitality with fewer apoptotic cells compared to those without EVs and showed elevated metabolic activities after 5 days. This way, a successful system was created with the ability to influence several cardiac regeneration processes, such as CM proliferation, reduction of cell death, and possessing angiogenic potential (Bar et al., 2023).

**FIGURE 3:**
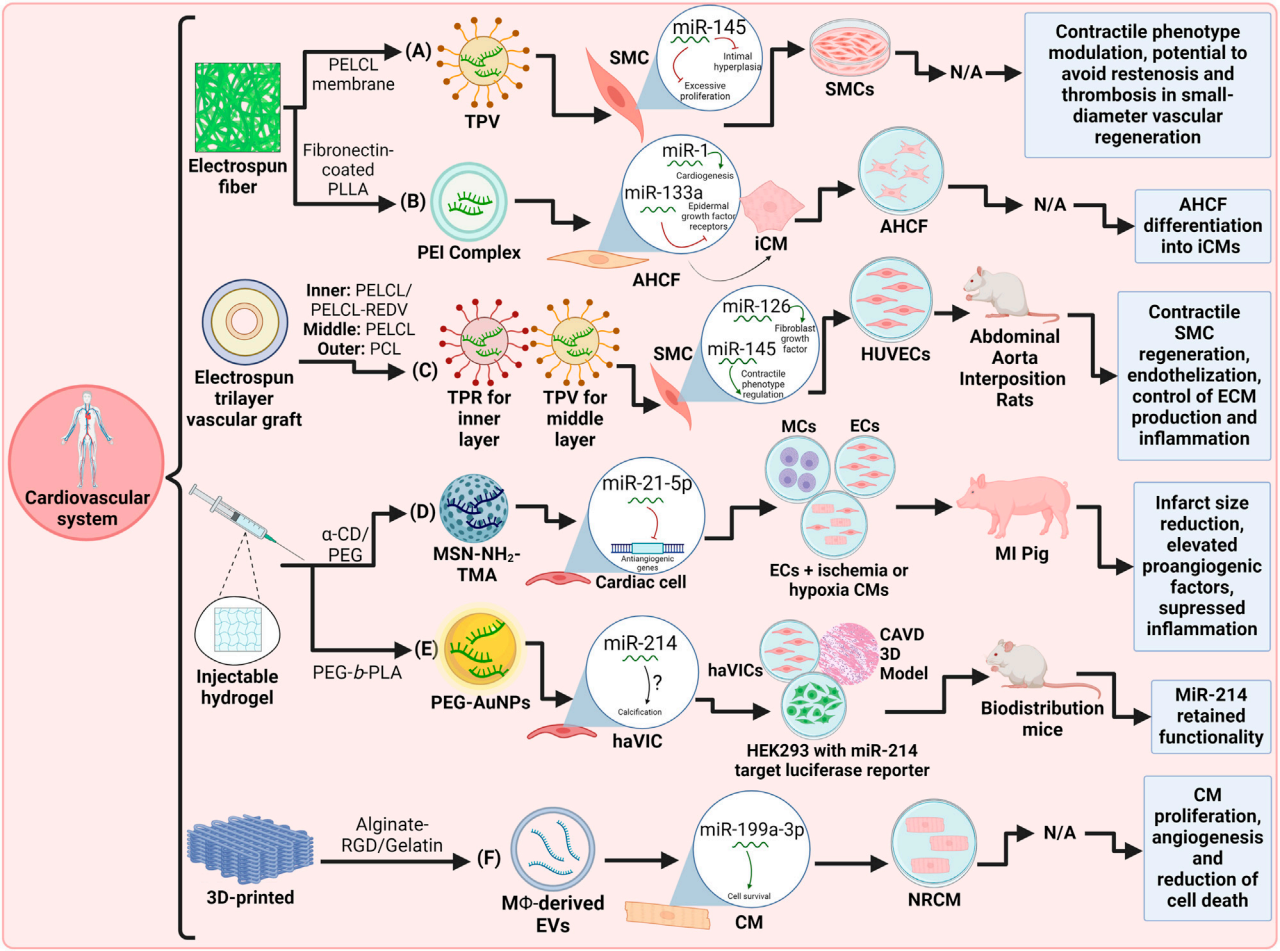
Schematic representation of recent miRNA-integrated cardiovascular system tissue engineering strategies: **(A)** An electrospun PELCL fiber membrane with miR-145-loaded TPV for target-regulating SMCs. (Wen et al., 2019). **(B)** A fibronectin-coated electrospun PLLA fiber scaffold with miR-1 and miR-133a complexed to PEI for cardiac fibroblast transdifferentiation (Muniyandi et al., 2021). **(C)** An electrospun trilayer vascular graft with miR-126-loaded TPR and miR-145-loaded TPV for small-diameter vascular regeneration (Wen et al., 2020). **(D)** An α-CD/PEG injectable hydrogel with miR-21-5p-loaded MSN-NH2-TMA for myocardial infarction therapy (Li et al., 2021). **(E)** A PEG-b-PLA injectable hydrogel with miR-214-loaded PEG-AuNPs for CAVD (Van Der Ven et al., 2021). **(F)** An alginate-RGD/gelatin 3D-printed scaffold with miR-199a-3p-loaded MΦ-derived EVs for cardiac patches (Bar et al., 2023). Created with Biorender.com.

In summary, scaffold-mediated miRNA delivery has considerably advanced tissue engineering, especially in CVDs, as depicted in Figure 3. This approach offers targeted therapy for conditions like MI and HF. Proper scaffold integration is achieved by functional scaffolds like PELC and PLLA electrospun membranes, trilayered vascular grafts, injectable hydrogel matrix, and PNP hydrogels. Studies show promising results, from advances in 3D-bioprinted CPs to advances in small-diameter vascular regeneration, mitigating inflammation post-MI, intimal hyperplasia, restenosis, or calcification, with therapeutics including rapid endothelialization, CM survival and proliferation, and regulation of circumferentially organized SMCs in the contractile phenotype. Nonetheless, these studies remain with some limitations and opportunities. Further, *in vivo* evaluations might be needed to evaluate the bioactivity of TPV for the regulation of SMCs. The PELC inner layer of the TEVG produced visible calcification; therefore, well-rounded approaches that take into account the properties of biocompatible materials are desirable, like the use of natural polymers in conjunction with composite multilayered scaffolds. More research is crucial to determine whether the Gel@MSN/miR-21-5p strategy is effective in chronic instances and whether there is an ideal therapeutic time frame, as immediate treatment following an MI may not be applicable in practical settings. The *in vivo* evaluation of PEI-miR-1 and PEI-miR-133a polyplexes is still unexplored. Findings on the effect of AuNP-miR-214 provide more evidence that miR-214 has a role in the development of CAVD (via promoting ALP), encouraging further investigation. Nonetheless, concerns remain over the potential harm that innate EV cargo may cause to recipient cells outside of the 3D-bioprinted patch. However, these advancements indicate a transformative shift towards precision medicine in cardiovascular care, with the potential to revolutionize treatment strategies and improve patient outcomes.

## 5 MiRNA-integrated skin tissue engineering strategies

In recent years, the challenge of promoting tissue regeneration in various skin defects, including large traumatic injuries, burns, bedsores, and chronic diabetic ulcers, has emerged as a significant clinical concern (Tottoli et al., 2020; Hama et al., 2023). Unfortunately, traditional methods like skin grafts have limitations, including donor site morbidity, graft complication, and limited availability (Ke et al., 2023; Šuca et al., 2024). Remarkably, tissue engineering has shown great potential in treating skin wounds. Along with sustainable materials like collagen, cellulose, and chitosan, these alternative therapies not only enhance wound healing and lower infection risk but also help reduce inflammation and stimulate new blood vessel growth (Elfawy et al., 2023; Hosseinpour et al., 2024). Moreover, the delivery of miRNAs through skin tissue engineering scaffolds presents a promising approach to enhancing skin healing. By incorporating specific miRNAs into these scaffolds, researchers can modulate gene expression at the wound site, promoting cellular processes essential for tissue repair, such as cell proliferation, migration, and angiogenesis (Dey et al., 2023; Guelfi et al., 2024).

A study conducted by Saleh et al. (2019) demonstrated the acceleration of wound healing by adhesive GelMA-based hydrogel containing miR-223 mimic since the overexpression of miR-223 was previously proved to induce the polarization of MCs towards the anti-inflammatory (M2) phenotype, perhaps facilitating the acceleration of wound healing, while the hydrogel protects the poorly stable miR-223 mimic. In this study, hyaluronic acid NPs (to regulate the polarization of tissue MCs during wound restoration), loaded with miR-223-5p mimic, were embedded in the GelMA hydrogel, which was placed above the wound. GelMA-based hydrogels were selected due to their ability to attach, infiltrate, and proliferate cells that help to mediate wound re-epithelialization and healing. *In vitro* studies showed that the overexpression of miR-223 mimicked J774A.1 MCs elevate the levels of the anti-inflammatory gene Arg-1 and lessen the levels of pro-inflammatory markers, including TNF-α, IL-1β, and IL-6. Likewise, *in vivo* experiments demonstrated that the adhesive hydrogels effectively adhered to and covered the wounds throughout the healing process in a mice model of acute excisional wounds.

Other miRNAs and gels have also been used for similar purposes. For instance, a non-cytotoxic biomaterial system of topically applicable or injectable self-healable zwitterionic cryogels with miR-146a-cerium oxide NPs (CNPs) was demonstrated to reduce inflammation and improve wound healing in diabetic patients (Sener et al., 2020). CNPs were specifically chosen due to their ability to eliminate oxidative stress and balance the oxidant-to-antioxidative enzyme ratio in diabetic wounds. Zwitterionic cryogels were created using either [2-(methacryloyloxy) ethyl]dimethyl-(3-sulfopropyl) ammonium hydroxide (SBMA) or 3-[[2-(methacryloyloxy) ethyl] dimethylammonio] propionate (CBMA) as zwitterionic monomers along with hydroxyethyl methacrylate (HEMA) as a non-zwitterionic crosslinker. Moreover, the gel was tested *in vivo* on old female mice with diabetic wounds and the sustained release of miR-146a CNPs from these gels was evidenced to speed up diabetic wound healing time and significantly reduce inflammation; furthermore, qPCR results showed the downregulation of pro-inflammatory cytokines, such as IL-6 and chemokine ligand 2 (CXCL2), as well as the upregulation of col-I gene.

Likewise, Yang et al. (2021) delivered a small interfering RNA 29a (siRNA-29a) through a hydrogel to downregulate miR-29a among diabetic patients since the underexpression of this miRNA has been observed to enhance angiogenesis and col-I synthesis, thereby boosting wound healing. This gel was composed of oxidized hydroxymethyl propyl cellulose (OHMPC) and adipic dihydrazide-modified HA (HA-ADH), which are known for having good biocompatibility and being easy to modify. SiRNA-29a was loaded onto a HA-PEI complex and embedded on the hydrogel along with the anti-inflammatory phytochemical oridonin (ori), which was loaded onto alginate microspheres (Alg), resulting in an OHMPC/HA-ADH/Alg@ori/HA-PEI@siRNA-29a hydrogel system. The system was tested *in vitro* on mouse fibroblasts L292 cell cultures to demonstrate biocompatibility and *in vivo* on STZ-induced type I diabetic SD rats to verify wound healing. The outcome was promising; the hydrogel system accelerated wound healing among diabetic SD rats by potentially inhibiting pro-inflammatory factors such as IL-6 and TNF-alpha, and complete epidermal coverage was achieved within 3 weeks of post-treatment.

In another study, Hu et al. (2021) used PLGA-LIP electrospun fiber to deliver miR-145 and platelet-derived growth factor composed of two B subunits (PDGF-BB) synergistically for wound healing promotion. The miR-145 and PDGF-BB were selected due to their crucial role in the differentiation of MSCs into vascular smooth muscle cells (VSMCs), therefore aiding in the process of wound healing. In this study, the miR-145 and PDGF-BB were loaded onto biodegradable LIPs formed by the cationic lipid DOTAP (N-[1-(2,3-dioleoyloxy) propyl]-N,N,N-trimethylammonium methyl-sulfate) to prevent a premature drug release and achieve a higher healing effect. MSCs were the main target in this investigation because these cells are able to promote tissue repair through their autocrine ability. An *In vitro* study using HUVECs confirmed the promotion of the tubular formation of ECs through the visualization of angiogenesis of different fibrous scaffolds, and interestingly, the combination of PLGA-LIP with both miR-145 and PDGF-BB resulted in a significantly greater total length of the tube and number of junctions compared to PLGA-LIP engaged with either miR-145 or PDGF-BB alone. On the other hand, *in vivo* testing was carried out on SD rats to estimate the protective effect of PLGA-LIP engaged with or without miR-145 or PDGF-BB on skin wound healing, and the results revealed a significant additive protective effect on angiogenesis and wound healing when PLGA-LIP was engaged with both miR-145 and PDGF-BB.

Relatedly, Mulholland et al. (2022) created a peptide/miR-31 nanomedicine within an electrospun material designed to regenerate wounds *in vivo*. The miR-31 was chosen since it has been shown to be underexpressed in conditions related to slow wound healing. The miRNA delivery system used a plasmid encoding miR-31 (pmiR-31) encapsulated by the linear cell-penetrating peptide (CPP), CHAT, via a wound patch composed of electrospun polyvinyl alcohol (PVA) nanofibers, with the purpose of improving cellular entry and trigger miR-31 expression *in vitro* in both human keratinocyte cell line (HaCaT) and human microvascular EC line HMEC-1. *In vivo* experiments were performed on live C57BL/6 J mice, where CHAT/pmiR-31 was administered via electrospun PVA nanofibers. The results showed a significant augmentation in the epidermal layer and the stratum corneum layer compared to the control group. In addition, *in vivo* therapy with CHAT/pmiR-31 resulted in enhanced angiogenesis in wounds as compared.

Recently, Li et al. (2023) developed engineered exosomes containing miR-146a and SF patch (SFP) to promote diabetic wound healing by interfering with interleukin-1 receptor-associated kinase 1(IRAK1), which has been associated with the NF-κB inflammation signaling pathway. The SF binding peptide (SFBP) was selected via phage display. Subsequently, a fusion protein called SFBP-Gluc-MS2 (SGM) and a pac-miR-146a-pac fusion protein were synthesized. The constructed exosome SGM-miR-146a-exos was obtained from placental MSCs (PMSCs) that were transduced with SGM and pac-miR-146a-pac protein. SF was chosen for its antimicrobial properties to prevent pathogen invasion, thereby reducing the risk of wound infection, while miR-146a was shown to act as an anti-inflammatory regulator. *In vivo* experiments with BKS-DB (db/db) mice showed that SGM-miR-146a-Exo significantly accelerated the wound healing process while it decreased the IRAK1 and IL-6 expressions, resulting anti-inflammatory effect.

**FIGURE 4:**
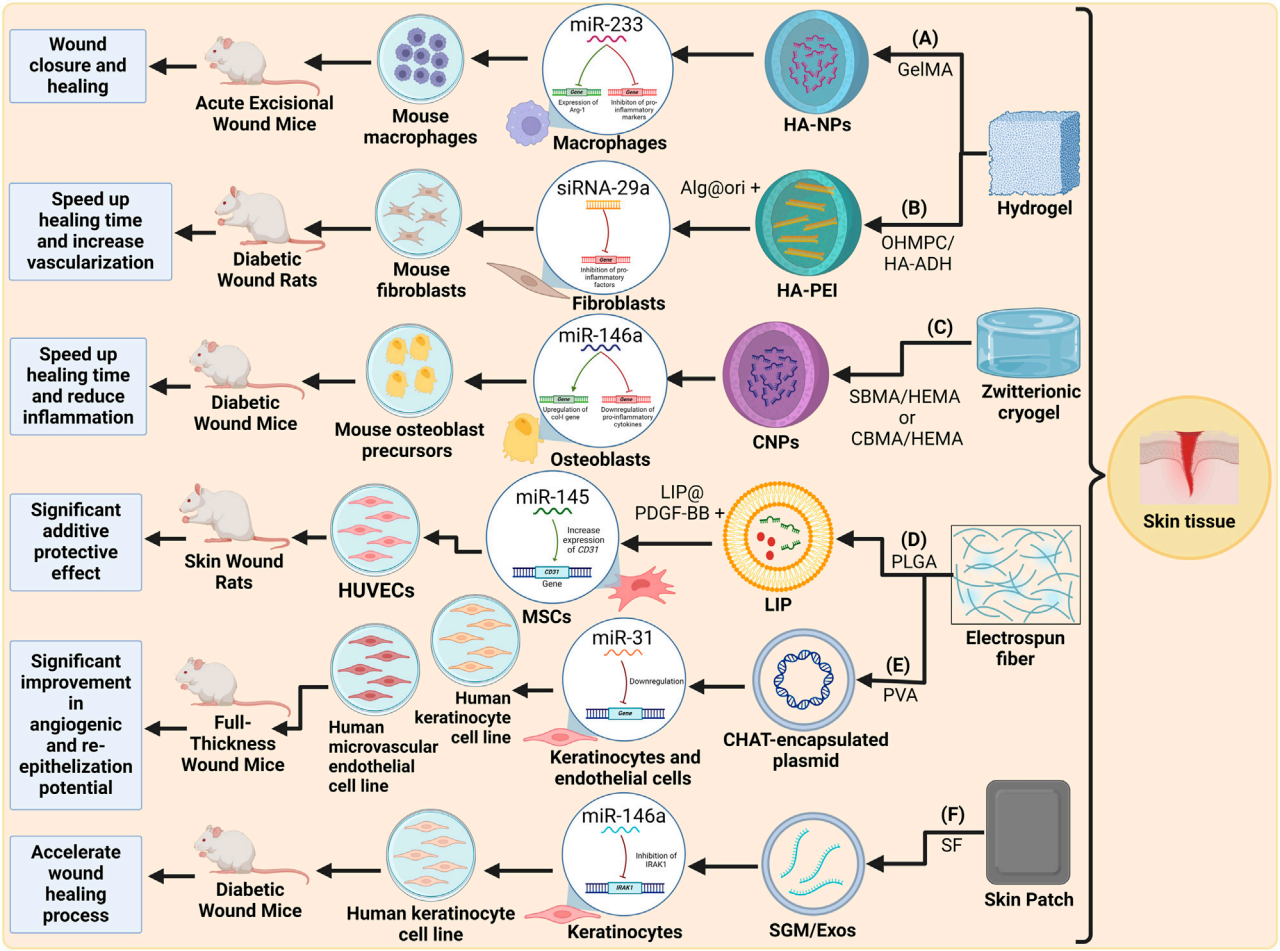
Schematic representation of recent miRNA-integrated skin tissue engineering strategies: **(A)** A GelMA hydrogel scaffold with miR-233-loaded HA-NPs for local immunomodulation of wounds (Saleh et al., 2019). **(B)** A OHMPC/HA-ADH hydrogel scaffold with siRNA-29a-loaded HA-PEI and Alg@ori for chronic diabetic wound healing (Yang et al., 2021). **(C)** A zwitterionic cryogel with miR-146a-loaded CNPs for wound healing (Sener et al., 2020). **(D)** An electrospun PLGA fiber scaffold with miR-145-loaded LIP and PDGF-BB-loaded LIP for wound healing (Hu et al., 2021). **(E)** An electrospun PVA fiber scaffold with miR-31-containing plasmids encapsulated in CHAT for wound healing (Mulholland et al., 2022). **(F)** A SF skin patch with miR-146a-loaded SGM/Exos for diabetic wound healing (Li et al., 2023). Created with Biorender.com.

In conclusion, it could be stated that skin tissue engineering technology has been advancing efficiently to eventually save or improve people’s lives, especially patients with acute burn wounds or chronic diabetic wounds, as portrayed in Figure 4. However, there are still some challenges that should be overcome, for example, low stability of the miRNAs, low transfection efficiency and the delicacy of the porous on the hydrogels that can lead to decreases in the final outcome of the scaffold applied. Taking all of these challenges into account, it is evident that miRNAs can be a valuable part of scaffold-based technologies for improving the chronic or acute skin wound healing process when induced effectively.

## 6 MiRNA-integrated muscular and connective tissue engineering strategies

Skeletal muscles and connective tissues are indispensable for health. Nevertheless, as life expectancy increases, so do musculoskeletal disorders due to a significant loss of muscle mass commonly seen with aging. Cachexia, atrophy, osteoarthritis, and osteoporosis are some of the main diseases related to muscle dysfunction that affect a person’s quality of life. Several factors are involved in the pathogenesis of muscle wasting, such as senescence, impaired muscle regeneration, inflammation, apoptosis and oxidative stress. On the other hand, molecular mechanisms such as terminal differentiation into myocytes and their fusion into myofibers, activation of quiescent satellite cells and proliferation of myoblasts (myogenic precursor cells) are needed for proper muscle regeneration. miRNAs play an important role as regulators of skeletal muscle function and it has been demonstrated that the aberrant expression of miRNAs significantly impacts the progression of muscle atrophy. For instance, the upregulation of miR-29b is usually seen in different types of muscle dysfunction. Other miRNAs participate in myogenesis by regulating proliferation, myoblast differentiation and satellite cell quiescence (Brzeszczyńska et al., 2020).

An effective tissue regeneration needs a prolonged exposure to bioactive molecules, yet approaches for their delivery like high dosage or systemic intravenous administration, generate unwanted effects in other tissues. At the same time, current treatment methods present a limited regeneration potential. Therefore, novel strategies have emerged, implementing scaffold-mediated delivery and miRNA-based therapeutics to regulate the expression of specific genes by manipulating the RNA interference pathway. The scaffold-mediated delivery of miRNAs presents significant advantages over other methods because of its prolonged activity and controlled, sustained, and bioresponsive effect (Kelly et al., 2019; Dasgupta and Chatterjee, 2021).

Feng et al. (2018) proposed a two-stage delivery system of therapeutic miRNA as a novel, sustainable, and bioresponsive method for chronic intervertebral disc degeneration (IDD) treatment. The overexpression of matrix metalloproteinases (MMPs) is responsible for the main pathological features of IDD since it leads to the degradation of the ECM and the progressive fibrosis invasion that causes the degeneration of the nucleus pulposus. In this study, miR-29a was chosen as the therapeutic miRNA for its powerful antifibrotic capability by silencing MMP-2 expression and blocking the β-catenin translocation pathway from the cytoplasm to the nucleus. The miRNA delivery mechanism implements MMP-responsive cationic block copolymers, poly (ethylene glycol)-GPLGVRG-poly{N′-[N-(2-aminoethyl)-2-aminoehtyl]aspartamide}-cholesteryl (PEG-GPLGVRG-PAsp(DET)-Chole) (PGPC), loading miR-29a for its degradable properties, high gene transfection efficiency and exceptional endosomal escape ability. To obtain a sustained release, the polyplex micelles were encapsulated into MMP-responsive PEG/CGPLGVRGC injectable hydrogels, where the peptide linkage (GPLGVRG) is specifically cleaved by MMP enzymes. *In vivo* experiments were conducted using animal models with chronic IDD in order to assess the therapeutic potential of the two-stage delivery system proposed. Sprague-Dawley rats were used for *in vivo* delivery analysis and New Zealand white rabbits were employed to evaluate *in vivo* fibrosis inhibition. The results in the first stage showed a successful and localized release of miR-29a polyplex micelles in diseased tissues, thanks to MMP-responsive degradation of hydrogels triggered by the elevated MMP levels present in intervertebral disc (IVD) regions. Likewise, the second stage showed an efficient endosomal escape and enhanced cellular internalization into nucleus pulposus cells due to the MMP-responsive dePEGylation of the micelles. This novel strategy allows a bioresponsive and sustained two-stage delivery of miR-29a into the target cells, achieving a significant attenuation of fibrosis progression in IVD (Feng et al., 2018).

Treatment options for damaged articular cartilage caused by osteoarthritis or trauma are deficient and limited. Addressing this issue, Lolli et al. (2019) present a novel strategy to enhance *in situ* cartilage regeneration by endogenous cells, using FB/HA hydrogel-based delivery of a locked nucleic acid miRNA that silences miR-221 (antimiR-221). miR-221 plays an important role in osteochondral repair due to its anti-chondrogenic effect; therefore, antimiR-221 chemically modified to contain locked nucleic acid (LNA) bases and a phosphothioate backbone (which allowed for cellular intake) was used in this study to inhibit miR-221 and induce chondrogenesis. FB/HA conjugate hydrogels were employed because of their pronounced stability and heightened viscoelastic and mechanical properties. *In vitro* experiments were carried out to assess the retention of antimiR-221 by FB/HA hydrogels and to evaluate the transfection of human MSCs (hMSCs) in antimiR-loaded FB/HA hydrogels. For the *in vivo* studies, a female NMRI nu/nu mouse (osteochondral defect model) was used to investigate the capability of antimiR-221 loaded FB/HA hydrogels for cartilage production by endogenous cells. The main findings were that FB/HA hydrogels effectively enable the transfection of antimiR-221 into hMSCs, as well as the knockdown of miR-221 *in situ*, leading to the stimulation of chondrogenesis. This study demonstrates that hydrogel-assisted anti-miRNA delivery successfully enhanced endogenous cartilage repair *in vivo*, establishing it to be a promising miRNA therapy for tissue repair.

Novel strategies for tendon repair have been developed involving tissue engineering and 3D printing technology. Wu et al. (2021) suggest a miRNA-based RNAi (RNA interference) plasmid loaded on poly-dopamine nanoparticles (PDA-NPs) embedded on a 3D tendon scaffold as a new treatment method that prevents tendon adhesion and leads to proper tendon repair. The miRNA used in this study silences the transforming growth factor β-1 (*TGF-β1)* gene, which decreases the incidence of tendon adhesions. A 3D tendon scaffold was implemented for its optimal biocompatibility and sustained release of the plasmid. In order to assess the effect of the *TGF-β1* gene silencing plasmid-loaded tendon scaffold *in vivo*, a chicken tendon defect model was employed. The results showed that the 3D tendon scaffold loaded with *TGF-β1* gene silencing plasmid was able to effectively regenerate the tendon and recover its biofunction by preventing the appearance of tendon adhesion.

Seeking to enhance the quality of seed cells in order to augment the effectiveness of cartilage restoration, Shen et al. (2022) implemented an injectable SF hydrogel containing articular chondrocytes (ACs) and hypoxia preconditioned exosomes (H-Exos) derived from BMSCs to enhance cartilage regeneration *in vivo*. The hypoxia preconditioning achieved an upregulated expression of miR-205-5p in H-Exos, significantly stimulating the migration, anabolism, proliferation and anti-inflammatory effects of ACs through the miR-205-5p/PTEN/AKT pathway. The SF hydrogel was used because of its ability to preserve and sustainably release H-Exos. *In vivo* experiments were executed using mice as the cartilage defect model. The results revealed the biological efficacy of the injectable SF hydrogels and efficient promotion of the functions of ACs by H-Exos, as well as the successful activation of the PTEN/AKT pathway by the upregulation of miR-205-5p.

Severe skeletal muscle injuries commonly present an inefficient regeneration process due to the formation of scar tissues, significant myofiber loss, and serious muscle function impairment. In this context, Song et al. (2022) projected a novel therapeutic strategy implementing a silk sericin patch delivering miR-29-enriched EVs-decorated myoblasts (SPEED) that promotes functional repair and regeneration in severe skeletal muscle injuries. miR-29-enriched EVs (miR-29-EVs) were used for its effectiveness in delivering miR-29 into primary myoblasts. MiR-29 plays an important role as a positive regulator of myogenesis since it increases the expression of myogenic genes while simultaneously suppressing the transdifferentiation of C2C12 myoblasts into myofibroblasts by inhibiting the expression of fibrotic genes. In this study, a silk sericin patch was selected due to its biodegradability, relatively low immunogenicity, natural cell-adhesive feature, and degradation time that allows myoblasts fusion and proper bioactivity of the scaffold. Additionally, *In vivo* trials were carried out employing a severe mouse tibialis anterior (TA) skeletal muscle injury model, and the results revealed that the sericin patch was capable of supporting the adhesion and proliferation of myoblasts; miR-29-EVs achieved an efficient differentiation of myoblasts into myotubes, instead of myofibroblasts; and that SPEED was able to promote the integration of primary myoblasts into host muscle. These findings demonstrate that the implantation of SPEED remarkably improved the muscular microenvironment, accomplishing a functional and structural recovery of severe skeletal muscle injuries.

**FIGURE 5:**
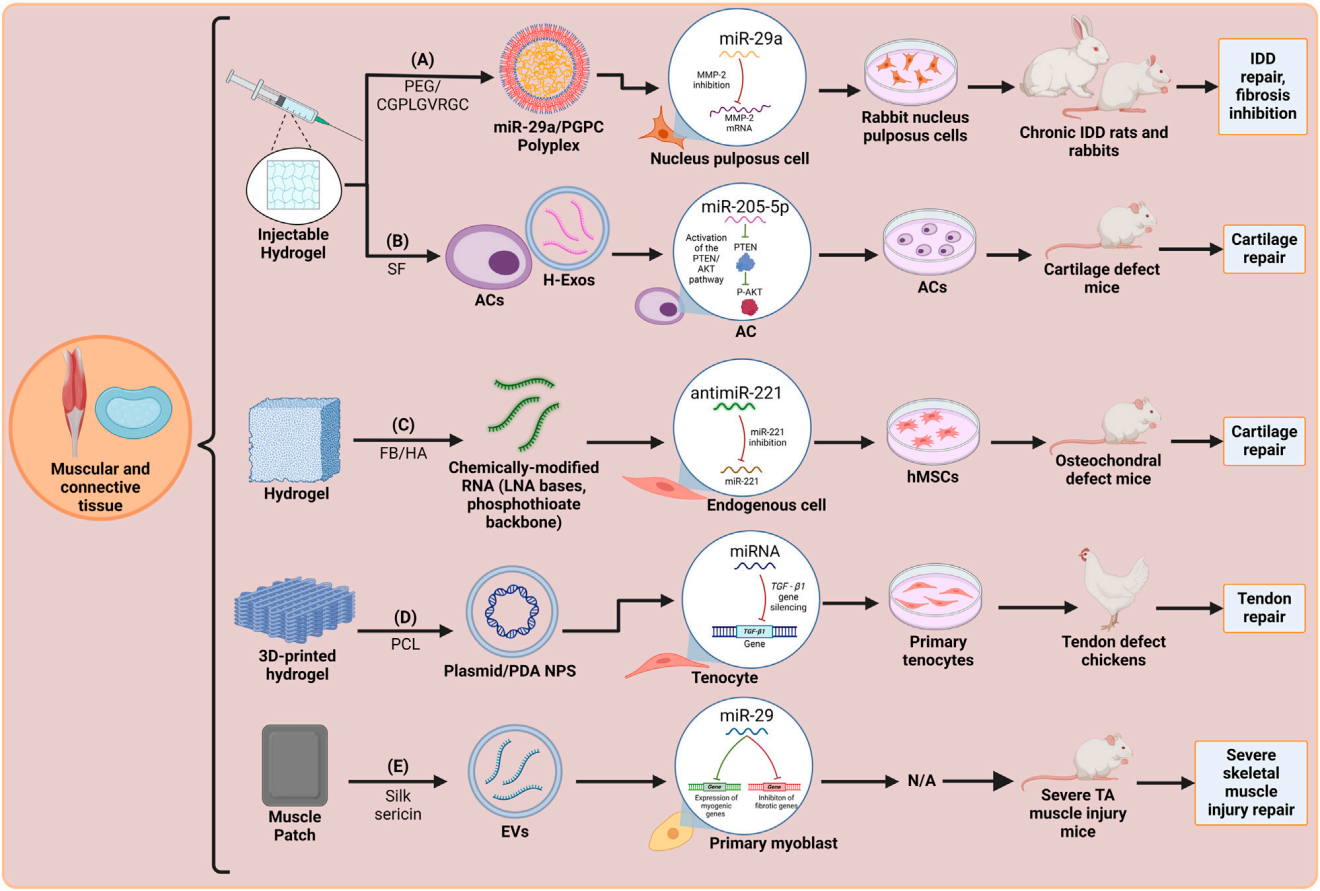
Schematic representation of recent miRNA-integrated muscular and connective tissue engineering strategies: **(A)** A PEG/CGPLGVRCC injectable hydrogel with miR-29a/PGPC polyplex for IDD repair (Feng et al., 2018). **(B)** A SF injectable hydrogel with miR-205-5p-loaded H-Exos and ACs for cartilage repair (Shen et al., 2022). **(C)** A FB/HA hydrogel scaffold with an antimir-221 sequence with LNA bases and phosphothioate backbone for cartilage repair (Lolli et al., 2019). **(D)** A PCL 3D-printed hydrogel scaffold with RNAi plasmid-loaded PDA NPs for tendon repair (Wu et al., 2021). **(E)** A silk sericin muscle patch with miR-29-loaded EVs for severe skeletal muscle injury repair (Song et al., 2022). Created with Biorender.com.

In conclusion, both *in vitro* and *in vivo* experiments have demonstrated that miRNAs are the key regulators of muscle atrophy (Figure 5) and that some promising miRNAs now need to be evaluated in human muscle disorders clinical trials. Additionally, it is important to highlight the strong therapeutic potential of scaffold-mediated miRNA delivery systems for muscle-related dysfunction and atrophy. Nevertheless, in order to improve the efficacy of miRNA treatment methods, future studies should be carried out to identify new targeting ligands and to characterize disease-specific markers on tissues. Furthermore, most of the *in vivo* experiments are performed in quadrupedal models, not taking into consideration the differences between quadrupedal and bipedal (human) species. Also, comprehending the processes that lead to miRNA dysregulations will help to recognize prime targets in disease pathogenesis and implement therapeutic and predictive biomarkers, as well as preventive therapies. Finally, more effort needs to be placed on finding new, sustained and bioresponsive scaffold materials that enhance miRNA delivery.

## 7 Other miRNA-integrated tissue engineering strategies

Zhao et al. (2018) tested a collagen-based material combined with miRNA with the objective of repairing the cornea and inhibiting scar formation. The scaffold was made from nanocomplexes of AuNPs and miR-133b. AuNP/miR-133b complexes were loaded into collagen using two different methods, such as surface loading (Col-AMS) and inside loading (Col-AMI). The evaluation of the scaffold took place *in vitro* and *in vivo*. To evaluate the cytocompatibility of AuNPs and the ability of miR-133b to inhibit myofibroblast transformation, rabbit corneal stromal cells were used. On the other hand, *in vivo* tests were performed in a mouse model to verify the cornea epithelialization process. It was observed that the scaffold was able to downregulate the expression of myofibroblast transformation genes (alpha-smooth muscle actin and type 1 collagen) in corneal stromal cells. In addition, the corneas transplanted with Col-AMS and Col-AMI were functioning similarly to healthy corneas. With this outcome, it was established that collagen membranes combined with AuNP/miR-133b complexes can rapidly repair corneas and effectively inhibit scar formation.

The following year, Rahmani et al. (2020) evaluated the impact of hsa-miR-9-1 overexpression in photoreceptor differentiation of conjunctiva MSCs on a 3D nanofibrous scaffold. The aim was to find a new approach to guide and differentiate conjunctiva MSCs (CJMSCs) into photoreceptor-like cells by using hsa-miR-9-1 induction without neither chemical and biological growth factor nor external stimuli on both types of delivery: 2D and 3D substrate. The scaffold was made from SF-PLLA, prepared as a 3D microenvironment (due to the important role of 3D nanofibers in the differentiation of stem cells because of their similarity to the ECM), and fabricated by the electrospinning technique. Later, CJMSCs were transduced by a lentiviral vector carrying miR-9 (pCDH + has-miR-9-1) and seeded on both 2D tissue culture grade polystyrene (TCPS) and the scaffold. Afterward, the scaffold was tested, and it was noticed that more than 80% of CJMSCs were transduced and miR-9 expression was significantly higher in miR-9-CJMSCs compared to the empty vector.

A novel approach to ureteral tissue engineering (for urinary tract restoration) was made by Zhao et al. (2019), using a natural collagen material compatible with cell survival as a graft, known as vessel ECM (VECM), and differentiated urine-derived stem cells (USCs). These cells were harvested from human urine samples and cultivated in an induction medium, resulting in cell differentiation into smooth muscle and urothelium phenotypes. For the promotion of the contractile phenotype of the differentiated USCs into mature contractile SMCs, induced cells were transfected with a miR-199a-5p plasmid using a recombinant lentivirus-mediated gene transfer system and treated with transforming growth factor b-1 (TGF-b1). The combination of TGF-b1 and miR-199a-5p triggers the differentiated cells to express the smooth muscle phenotype gene and allows them to change into the contractile phenotype, which is essential for smooth muscle function. Additionally, the results showed that miR-199a-5p regulates cytoskeleton remodeling. Moreover, in this study, the dynamic culture technique was utilized for seeding cells onto VECM and encouraged their penetration into the 3D pores of matrix fibers *in vitro*, demonstrating sufficient biocompatibility. During dynamic culture, the induced smooth muscle cells formed 3 to 6 cell layers, while the induced urothelium cells formed a single epithelial layer. The tubular graft underwent vascularization following 3 weeks of omental maturation, and subsequently, in an *in vivo* rabbit model, the matured graft was employed for ureter restoration. Intriguingly, histological examination at 2 months revealed a distinctly stratified ureter with multilayered urothelium covering the well-organized smooth muscle tissue.

Another promising possibility that tissue engineering has to offer is bladder augmentation (enterocystoplasty) avoiding donor tissue morbidity. For instance, a tri-layer hydrogel scaffold composed of bladder acellular matrix graft-alginate di-aldehyde gelatin hydrogel-silk mesh (BAMG-HS) was created by Xiao et al. (2021) for encapsulation of EVs derived from human Adipose-derived stem cells (ASCs) as drug delivery vehicles for proangiogenic effector miR-126, with the aim to aid in bladder regeneration. BAMG-HS serves as a waterproof barrier that provides strong mechanical and biodegradable qualities, encourages angiogenesis and supports bladder morphological regenerations of neural, smooth muscle, and urothelium, as well as functional restoration without severe inflammation or fibrosis. *In vitro*, human ASCs-EVs increased the proliferation of HUVECs within a week and improved the HUVEC tube formation in a concentration-dependent way. An assessment was conducted using a rat bladder augmentation model. After 12 weeks, with the exception of inadequate innervation, ASCs-EVs enabled bladder abnormalities to regenerate fully. Encapsulating ASCs-EVs in BAMG-HS allowed for bladder gross morphological regeneration *in vivo* while reducing inflammation and fibrosis. Following HUVEC internalization, MiR-126 from human ASCs-EVs stimulated bladder regeneration angiogenesis by blocking RGS16, which activates the CXCR4/SDF-1α pathway and increases VEGF secretion through activation of ERK1/2 phosphorylation.

Interstitial renal fibrosis (RIF) could also be treated with a safe and effective intervention by sustained local delivery of therapeutic miRNA. The method was developed in 2021 by Xu et al. (2021) using miR-29b, which is demonstrated to be downregulated in renal fibrosis and is significantly implicated during the development of multiple organ fibrosis. NPs made from cationic bovine serum albumin (cBSA), which has been exhibited to precisely and dose-dependently block fibroblast activation *in vitro*, are used to create nanocomplexes (cBSA/miR-29b) by complexing with negatively charged miR-29b. A host-guest supramolecular hydrogel is then used to inject and deliver cBSA/miR-29b to the kidney. The hydrogel is achieved through host molecule β-cyclodextrin (β-CD) and the guest molecule adamantane (Ad) chemically modified, subsequently added to triblock copolymer Pluronic F127 and HA as scaffolding materials, creating a shear-thinning F127-HA hydrogel. Using the normal rat kidney interstitial fibroblast (NRK-49 F) cell line, an *in vitro* model of TGF-β-induced fibrogenesis was developed. The activated NRK-49 F cells’ level of α-SMA, a crucial biomarker for activated fibroblasts, was considerably downregulated in a dose-dependent way by the cBSA/miR-29b nanocomplex, suggesting that they could successfully prevent or reverse fibroblast activation. The hydrogel has also been shown to be biocompatible and did not interfere with normal liver or kidney functions *in vivo*. Interestingly, A single injection of the hydrogel containing cBSA/miR-29b dramatically downregulated fibrosis-related proteins and genes in mice with unilateral ureteral obstruction for up to 21 days. A summary of the main results of the applications of miRNA-integrated tissue engineering strategies is shown in Table 1.

**TABLE 1:** Summary of miRNA-integrated tissue engineering strategies.

Reference	Therapeutic molecules	Delivery strategy	Scaffold type	Scaffold material	*In vitro* models	*In vivo* models	Application
Skeletal system							
Lei et al. (2019)	MiR-222 ASP	MSN-S-S-NH_2_	Injectable colloidal hydrogel	PEG-PLGA-PNIPAM	hBMSCs	Mandibular defect rats	Innervated bone tissue regeneration
Moncal et al. (2019)	MiR-148b	MiRNA-transfected BMSCs	3D-printed hydrogel	Collagen-filled PCL/PLGA/HAp	rBMSCs	Calvarial defect rats	Bone regeneration
Abazari et al. (2020)	MiR-2861	Adenoviral vector	Nanofibrous scaffold	PLGA	Human iPSCs		iPSC osteogenic differentiation
Qi et al. (2021)	MiR-181a/b-1	Lentiviral vector	Nanofibrous scaffold	PLGA	Human AT-MSCs		MSC osteogenic differentiation
Gan et al. (2021)	Chol-miR-26	MiRNA cholesterol modification	3D-printed hydrogel	Functionalized PEG	Human MSCs	Calvarial defect rats	Bone regeneration
Hu et al. (2022)	MiR-23a-3p	hUC-MSCs-sEVs	3D-printed hydrogel	GelMA/Nanoclay	Mouse BMSCs	Calvarial defect mice	Bone regeneration
Castaño et al. (2023)	MiR-210 Antagomir-16	HAp-NPs	Hydrogel	Collagen	Human MSCs	Calvarial defect rats	Bone regeneration
Limlawan et al. (2023)	MiR-302-a-3p	HAp-NPs-APTES	3D-printed hydrogel	TCA/HAp	HmOBs	Calvarial defect mice	Bone regeneration
Nervous system							
Milbreta et al. (2019)	MiR-219 MiR-338 NT-3	TKO	Fiber-hydrogel	PCLEEP/Col-1	Rat OPCs	SCI rats	SCI
Zhang et al. (2019)	MiR-21 MiR-132 MiR-222 MiR-431 NT-3	TKO	Fiber-hydrogel	PCL/Col-1	Rat cortical neurons DRGs	SCI rats	SCI
Nazari et al. (2020)	MiR-338	Lentiviral vector	Hydrogel	Fibrin	Human fibroblast iPSCs		iPSC differentiation into glial and neural progenitors
Jedari et al. (2020)	MiR-7	Lentiviral vector	Nanofibrous scaffold	PLLA/PCL	TMMSCs		MSC differentiation into oligodendrocytes (OL)
Zhang et al. (2021)	MiR-132 MiR-222 MiR-431 GDNF	TKO	Fiber-hydrogel	PCL/Col-1		SCI rats	SCI
Li et al. (2022)	MiR-222	CS-NPs	Nanofibrous scaffold	Poly-dopamine-coated SF	Mouse neuroepithelial cells		NSC differentiation into neurons
Liu et al. (2022)	MiR-21	CBD-LP-Exos	Hydrogel	Col-1		SCI rats	SCI
Wang et al. (2023)	MiR-29a	ZIF-8	NGC	SF/GT	Rat Schwann cells Rat adrenal medulla pheochromocytoma cells		PNI
Wan et al. (2023)	MiR-29a	PEG-SH-AuNPs	Nanofibrous scaffold	Self-assembling peptide	Rat NSCs	SCI rats	SCI
Cardiovascular system							
Wen et al. (2019)	MiR-145	TPV	Electrospun fiber membrane	PELCL	SMCs		Target-regulation of SMCs
Wen et al. (2020)	Inner layer MiR-126 Middle layer MiR-145	Inner layer TPR Middle layer TPV	Electrospun trilayer vascular graft	Inner layer PELCL/PELCL-REDV Middle layer PELCL Outer layer PCL	HUVECs	Abdominal aorta interposition rats	Small-diameter vascular regeneration
Li et al. (2021)	MiR-21-5p	MSN-NH2-TMA	Injectable hydrogel	α-CD/PEG	MCs ECs ECs and ischemia or hypoxia CMs	MI pig	Myocardial infarction
Muniyandi et al. (2021)	MiR-1 MiR-133a	PEI	Electrospun fiber	Fibronectin-coated PLLA	AHCF		Cardiac fibroblast transdifferentiation
Van Der Ven et al. (2021)	MiR-214	PEG-AuNPs	Injectable hydrogel	PEG-*b*-PLA	haVICs HEK293 with miR-214 target luciferase reporter CAVD 3D model	Biodistribution in mice	CAVD
Bar et al. (2023)	MiR-199a-3p	MΦ-derived EVs	3D-printed hydrogel	Alginate-RGD/gelatin	NRCM		Cardiac patches
Skin							
Saleh et al. (2019)	MiR-233	HA-NPs	Hydrogel	GelMA	Mouse macrophages	Acute excisional wound mice	Local immunomodulation of wounds
Sener et al. (2020)	MiR-146a	CNPs	Zwitterionic cryogel	SBMA/HEMA or CBMA/HEMA	Mouse osteoblast precursors	Diabetic wound mice	Wound healing
Yang et al. (2021)	SiRNA-29a Alg@ori	HA-PEI	Hydrogel	OHMPC/HA-ADH	Mouse fibroblasts	Diabetic wound rats	Diabetic wound healing
Hu et al. (2021)	MiR-145 PDGF-BB	LIP	Electrospun fiber	PLGA	HUVECs	Skin wound rats	Wound healing
Mulholland et al. (2022)	MiR-31	CHAT-encapsulated plasmid	Electrospun fiber	PVA	Human microvascular endothelial cell line Human keratinocyte cell line	Full-thickness wound mice	Wound healing
Li et al. (2023)	MiR-146a-	SGM/Exos	Skin patch	SF	Human keratinocyte cell line	Diabetic wound mice	Diabetic wound healing
Muscular and connective tissue							
Feng et al. (2018)	MiR-29a	PGPC polyplex	Injectable hydrogel	PEG/CGPLGVRCC	Rabbit nucleus pulposus cells	Chronic IDD rats Chronic IDD rabbits	IDD repair
Lolli et al. (2019)	Antimir-221	Modified RNA (LNA bases and phosphorothioate backbone)	Hydrogel	FB/HA	hMSCs	Osteochondral defect mice	Cartilage repair
Wu et al. (2021)	*TGF-β1* silencing miRNA	RNAi plasmid-loaded PDA NPs	3D-printed hydrogel	PCL	Primary tenocytes	Tendon defect chickens	Tendon repair
Shen et al. (2022)	MiR-205-5p	H-Exos and ACs	Injectable hydrogel	SF	ACs	Cartilage defect mice	Cartilage repair
Song et al. (2022)	MiR-29	EVs	Muscle patch	Silk sericin		Severe TA muscle injury mice	Severe skeletal muscle injury repair
Other tissues							
Zhao et al. (2018)	MiR-133b	AuNPs	Hydrogel	Collagen	Rabbit corneal stromal cells	Lamellar keratoplasty mice	Cornea repair
Rahmani et al. (2020)	Hsa-miR-9-1	Lentiviral vector	Nanofibrous scaffold	SF-PLLA	CJMSCs		MSC differentiation into photoreceptor cells
Zhao et al. (2019)	MiR-199a-5p	MiRNA-transfected USCs	Decellularized rabbit abdominal aorta	VECM	USCs	Ureteral defect rabbits	Urinary tract restoration
Xiao et al. (2021)	Middle layer MiR-126	Middle layer ASCs-EVs	Tri-layer hydrogel scaffold	Inner layer BAMG Middle layer Alginate di-aldehyde-gelatin hydrogel Outer layer SF mesh	HUVECs	Bladder augmentation rats	Bladder repair
Xu et al. (2021)	MiR-29b	cBSA NPs	Injectable hydrogel	F127-HA	Rat kidney interstitial fibroblast cell line	Unilateral ureteral obstruction mice	RIF

## 8 Concluding remarks

While the intersection between tissue engineering and miRNA-based therapeutics is still in development, the current review reveals that promising advances have been made over the last couple of years. The different techniques and strategies that have been studied have contributed to building a diverse and extensive toolkit for regenerative medicine and consolidating miRNAs as highly valuable biomolecules to employ in tissue engineering strategies. Nevertheless, reproducibility is still a challenge in this field and, although they are quite consistent with previous studies, the wide array of choices when it comes to scaffold fabrication and even testing models makes it hard to compare the results with one another. Additionally, clinical translatability for these combination approaches still seems far as none of the papers discussed the clinical trials and while *in vitro* and *in vivo* models are extremely valuable to learn about the safety and functionality of scaffolds and miRNA, significant differences may arise when translating. Nevertheless, the scientific studies discussed in this review display that this field is quite challenging, and several innovative strategies are being employed to overcome them and added implementations further enhance the functionality and precision of the works. Indeed, the knowledge of the intersection between miRNA-based therapeutics and tissue engineering holds its promise as it grows.

## 9 Future insights

The studies included in this review might provide insight into future miRNA-integrated tissue engineering strategies. Moreover, clinical trials are an intriguing frontier that has yet to be crossed in this subject, and they will hopefully be performed and analyzed in the near future. Indeed, getting to clinical trials would also require further insights into the safety and reproducibility of these strategies, potentially featuring studies looking into the long-term effects of miRNA-delivering scaffolds. Therefore, further experiments are still required in order to lay the groundwork for miRNA-integrated tissue-engineered scaffolds to enter the clinical panorama. Particularly, ensuring that the miRNAs used in miRNA-incorporated tissue-engineered scaffolds are specific to the desired pathway is crucial due to their pleiotropic nature, where a single miRNA can influence multiple targets and signaling pathways (Abbas et al., 2020; Ouyang and Wei, 2021). This specificity is vital to avoid unintended effects that could disrupt other cellular processes, potentially leading to adverse outcomes (Seyhan, 2024). Accordingly, by carefully selecting miRNAs that precisely target the intended pathways, the therapeutic efficacy of the scaffold could be enhanced, promoting the desired tissue regeneration while minimizing off-target effects.

In the future, miRNA-loaded tissue-engineered scaffolds can be further optimized by incorporating additional therapeutic agents such as phytochemicals, NPs, or approved drugs to enhance their efficacy. For instance, phytochemicals, known for their anti-inflammatory and antioxidant properties (Muscolo et al., 2024), could synergize with miRNAs to promote a more robust healing response, as it has been previously reported in other diseases like cancer (Javan et al., 2019; Ashrafizadeh et al., 2020). As a matter of fact, it has been evidenced that a scaffold of PVA nanofibers loaded with a flaxseed extract (rich in phytochemicals) can promote bone regeneration (Abdelaziz et al., 2024). Therefore, there is a large area of opportunity to evaluate the cooperative effects of miRNAs administered together with phytochemicals via scaffolds in tissue engineering. Nanoparticles, on the other hand, might be included in the design of tissue-engineered scaffolds to improve the stability and targeted delivery of miRNAs, ensuring they reach specific cellular compartments with greater precision (Leng et al., 2020; Bravo-Vázquez et al., 2023). On the whole, these combinatory strategies may provide a prospective avenue for advancing the effectiveness of miRNA-loaded scaffolds in tissue engineering and regenerative medicine.

It is worth mentioning that controlled release is vital for maintaining the stability and regulatory activity of miRNAs. In turn, an appropriate controlled release can ensure that miRNAs reach the target cells in their active form and increase their therapeutic potential (Moraes et al., 2021). In the case of miRNA-loaded tissue-engineered scaffolds, this controlled release may be optimized via advanced smart materials like temperature-sensitive hydrogels (Zhong et al., 2023), pH-responsive polymers and NPs (Leng et al., 2023), nanozymes (Wu et al., 2020), and biodegradable NPs (Anwar et al., 2021), all of which can be tailored to release miRNAs in response to specific environmental stimuli at the target site. The importance of considering the controlled release when developing miRNA-loaded tissue-engineered scaffolds was clearly evidenced by Pan et al. (2022), who observed that miRNA-activated hydrogel scaffolds generated by 3D printing and with high crosslinking degree displayed slow degradation rates and adequate release of miR-29b in the long term, thus stimulating bone regeneration. This research indicates that achieving an adaptable equilibrium between the scaffold’s degradation rate and the release pattern of miRNAs is crucial for effective tissue regeneration.

On the other hand, decellularized plant tissues might offer notable benefits as scaffolds for tissue engineering due to their 3D structure, which closely replicates the architecture of native tissues and creates an environment conducive to cell growth and tissue regeneration. While animal-derived decellularized tissues have been commonly used, they face challenges like limited supply, high costs, and ethical issues. In contrast, plant-based decellularized scaffolds provide a more accessible, cost-effective, and ethical alternative (Negrini et al., 2020; Harris et al., 2021). These scaffolds have demonstrated potential for supporting human cell growth, making them highly attractive for tissue engineering and other biomedical applications. However, more research is needed to fully understand how plant-based scaffolds impact cell behavior and response to external factors (Lacombe et al., 2020). Therefore, further exploring the applications of such plant-based tissues in conjunction with miRNAs could offer new avenues in the following years for regenerative medicine.

The standardization of protocols for miRNA-integrated tissue engineering strategies heavily depends on the type of targeted tissue. For instance, bone tissue may require different approaches compared to skin or neural tissues. Therefore, the first essential step is to standardize methods across tissues of the same nature. In this context, improving consistency among the properties and effectiveness of miRNA-loaded tissue-engineered approaches can be achieved by focusing on the common biological and mechanical characteristics within each tissue group. Besides, this tissue-specific standardization would benefit from the growing use of 3D bioprinting technology, which enables the precise fabrication of scaffolds (Beheshtizadeh et al., 2019). Also, the use of emerging 3D bioprinters might be helpful in optimizing important parameters involved in the production of engineered tissues, including bio-inks, feed rate, thickness, and air pressure (Rimann et al., 2016). Furthermore, as a starting point for standardizing the manufacture of tissue-engineered therapeutic products, the norms developed jointly by ISO and ASTM International (Additive Manufacturing Standards Development Framework) could be considered (Stanco et al., 2020).

Another fundamental step in optimizing miRNA-loaded tissue-engineered approaches must consist of assessing the reproducibility of their effects in a sufficiently large sample size to ensure reliable statistical competence (Abbas et al., 2021). Additionally, the effectiveness of these devices should be validated in other laboratories or medical centers to verify that their success is not tied to the specific conditions of the original research site. Remarkably, this is a major limitation within this field, as few studies have focused on ensuring the reproducibility of their positive effects on tissue regeneration. Likewise, the adoption of standardized outcome measurements can greatly facilitate the assessment of the efficacy of miRNA-loaded tissue-engineered devices and prompt their translation into the clinical landscape. Metrics like as the ATP/DNA ratio, scaffold cell viability, and efficacy in facilitating tissue regeneration are critical outcomes that should be evaluated during the characterization of these bioengineered products (Arora et al., 2020). Hence, homogenizing these outcomes may enhance comparability among different studies, particularly when examining the same tissue type or condition.

Unquestionably, the recent success of RNA-based therapeutics, particularly the mRNA-based vaccines against COVID-19, highlights the immense potential of RNA technology in modern medicine (Damase et al., 2021). This breakthrough paves the way for the development of other RNA-centered therapeutic approaches such as miRNA-loaded tissue-engineered scaffolds. As research continues to advance, these innovative scaffolds could revolutionize tissue healing and repair, offering precise, targeted therapies that harness the power of miRNAs to enhance the body’s natural regenerative processes. Hence, we believe that the content of this review will benefit the integration of miRNA technology into tissue engineering as a significant step forward in the evolution of next-generation medical treatments.

## References

[B1] Abazari M. F. Karizi S. Z. Kohandani M. Nasiri N. Nejati F. Saburi E. (2020). MicroRNA-2861 and nanofibrous scaffold synergistically promote human induced pluripotent stem cells osteogenic differentiation. Polym. Adv. Technol. 31, 2259–2269. 10.1002/pat.4946

[B2] Abbas N. Perbellini F. Thum T. (2020). Non-coding RNAs: emerging players in cardiomyocyte proliferation and cardiac regeneration. Basic Res. Cardiol. 2020 115 (5), 52–20. 10.1007/s00395-020-0816-0 PMC7398957 32748089

[B3] Abbas T. O. Elawad A. Pullattayil S. A. K. Pennisi C. P. (2021). Quality of reporting in preclinical urethral tissue engineering studies: a systematic review to assess adherence to the arrive guidelines. Animals 11, 2456. 10.3390/ani11082456 34438913 PMC8388767

[B4] Abdelaziz A. G. Nageh H. Abdalla M. S. Abdo S. M. Amer A. A. Loutfy S. A. (2024). Development of polyvinyl alcohol nanofiber scaffolds loaded with flaxseed extract for bone regeneration: phytochemicals, cell proliferation, adhesion, and osteogenic gene expression. Front. Chem. 12, 1417407. 10.3389/fchem.2024.1417407 39144698 PMC11322085

[B5] Adami G. Saag K. G. (2019). Osteoporosis pathophysiology, epidemiology, and screening in rheumatoid arthritis. Curr. Rheumatol. Rep. 21, 34–10. 10.1007/s11926-019-0836-7 31123839

[B6] Alonzo M. Primo F. A. Kumar S. A. Mudloff J. A. Dominguez E. Fregoso G. (2021). Bone tissue engineering techniques, advances, and scaffolds for treatment of bone defects. Curr. Opin. Biomed. Eng. 17, 100248. 10.1016/j.cobme.2020.100248 33718692 PMC7948130

[B7] Anesi A. Bartolomeo M.Di Pellacani A. Ferretti M. Cavani F. Salvatori R. (2020). Bone healing evaluation following different osteotomic techniques in animal models: a suitable method for clinical insights. Appl. Sci. 10, 7165. 10.3390/app10207165

[B8] Anwar M. Muhammad F. Akhtar B. (2021). Biodegradable nanoparticles as drug delivery devices. J. Drug Deliv. Sci. Technol. 64, 102638. 10.1016/j.jddst.2021.102638

[B9] Arora D. Babakhanova G. Simon C. G. (2020). Tissue engineering measurands. ACS Biomater. Sci. Eng. 6, 5368–5376. 10.1021/acsbiomaterials.0c00475 33320558

[B10] Ashrafizadeh M. Zarrabi A. Hushmandi K. Hashemi F. Moghadam E. R. Raei M. (2020). Progress in natural compounds/siRNA Co-delivery employing nanovehicles for cancer therapy. ACS Comb. Sci. 22, 669–700. 10.1021/acscombsci.0c00099 33095554 PMC8015217

[B11] Bar A. Kryukov O. Etzion S. Cohen S. (2023). Engineered extracellular vesicle-mediated delivery of miR-199a-3p increases the viability of 3D-printed cardiac patches. Int. J. Bioprinting 9 (2), 670. 10.18063/ijb.v9i2.670 PMC10090809 37065655

[B12] Barile A. Arrigoni F. Bruno F. Palumbo P. Floridi C. Cazzato R. L. (2018). Present role and future perspectives of interventional radiology in the treatment of painful bone lesions. Future Oncol. 14, 2945–2955. 10.2217/fon-2017-0657 29693420

[B13] Barile A. Arrigoni F. Zugaro L. Zappia M. Cazzato R. L. Garnon J. (2017). Minimally invasive treatments of painful bone lesions: state of the art. Med. Oncol. 34, 53–11. 10.1007/s12032-017-0909-2 28236103

[B14] Beaudart C. Biver E. Bruyère O. Cooper C. Al-Daghri N. Reginster J. Y. (2018). Quality of life assessment in musculo-skeletal health. Aging Clin. Exp. Res. 30, 413–418. 10.1007/s40520-017-0794-8 28664458 PMC5653197

[B15] Beavers K. R. Nelson C. E. Duvall C. L. (2015). MiRNA inhibition in tissue engineering and regenerative medicine. Adv. Drug Deliv. Rev. 88, 123–137. 10.1016/j.addr.2014.12.006 25553957 PMC4485980

[B16] Beheshtizadeh N. Lotfibakhshaiesh N. Pazhouhnia Z. Hoseinpour M. Nafari M. (2019). A review of 3D bio-printing for bone and skin tissue engineering: a commercial approach. J. Mater. Sci. 55 (9), 3729–3749. 10.1007/S10853-019-04259-0

[B17] Bravo-Vázquez L. A. Méndez-García A. Rodríguez A. L. Sahare P. Pathak S. Banerjee A. (2023). Applications of nanotechnologies for miRNA-based cancer therapeutics: current advances and future perspectives. Front. Bioeng. Biotechnol. 11, 1208547. 10.3389/fbioe.2023.1208547 37576994 PMC10416113

[B18] Brzeszczyńska J. Brzeszczyński F. Hamilton D. F. McGregor R. Simpson A. H. R. W. (2020). Role of microRNA in muscle regeneration and diseases related to muscle dysfunction in atrophy, cachexia, osteoporosis, and osteoarthritis. Bone Jt. Res. 9, 798–807. 10.1302/2046-3758.911.bjr-2020-0178.r1 PMC7672326 33174473

[B19] Cao J. Wu J. Mu J. Feng S. Gao J. (2021). The design criteria and therapeutic strategy of functional scaffolds for spinal cord injury repair. Biomater. Sci. 9, 4591–4606. 10.1039/d1bm00361e 34018520

[B20] Castaño I. M. Raftery R. M. Chen G. Cavanagh B. Quinn B. Duffy G. P. (2023). Dual scaffold delivery of miR-210 mimic and miR-16 inhibitor enhances angiogenesis and osteogenesis to accelerate bone healing. Acta Biomater. 172, 480–493. 10.1016/j.actbio.2023.09.049 37797708

[B21] Chandra P. K. Soker S. Atala A. (2020). Tissue engineering: current status and future perspectives. Princ. Tissue Eng., 1–35. 10.1016/b978-0-12-818422-6.00004-6

[B22] Damase T. R. Sukhovershin R. Boada C. Taraballi F. Pettigrew R. I. Cooke J. P. (2021). The limitless future of RNA therapeutics. Front. Bioeng. Biotechnol. 9, 628137. 10.3389/fbioe.2021.628137 33816449 PMC8012680

[B23] Dasgupta I. Chatterjee A. (2021). Recent advances in miRNA delivery systems. Methods Protoc. 4, 10. 10.3390/mps4010010 33498244 PMC7839010

[B24] Dellavia C. Canciani E. Pellegrini G. Tommasato G. Graziano D. Chiapasco M. (2021). Histological assessment of mandibular bone tissue after guided bone regeneration with customized computer-aided design/computer-assisted manufacture titanium mesh in humans: a cohort study. Clin. Implant Dent. Relat. Res. 23, 600–611. 10.1111/cid.13025 34139056

[B25] Dey A. D. Yousefiasl S. Kumar A. Moghaddam F. D. Rahimmanesh I. Samandari M. (2023). miRNA-encapsulated abiotic materials and biovectors for cutaneous and oral wound healing: biogenesis, mechanisms, and delivery nanocarriers. Bioeng. Transl. Med. 8, e10343. 10.1002/btm2.10343 36684081 PMC9842058

[B26] Diener C. Keller A. Meese E. (2022). Emerging concepts of miRNA therapeutics: from cells to clinic. Trends Genet. 38, 613–626. 10.1016/j.tig.2022.02.006 35303998

[B27] Ding W. Hu S. Wang P. Kang H. Peng R. Dong Y. (2022). Spinal cord injury: the global incidence, prevalence, and disability from the global burden of disease study 2019. Spine (Phila Pa 1976) 47, 1532–1540. 10.1097/brs.0000000000004417 35857624 PMC9554757

[B28] Doblado L. R. Martínez-Ramos C. Pradas M. M. (2021). Biomaterials for neural tissue engineering. Front. Nanotechnol. 3, 643507. 10.3389/fnano.2021.643507

[B29] Ehnert S. Rinderknecht H. Aspera-Werz R. H. Häussling V. Nussler A. K. (2020). Use of *in vitro* bone models to screen for altered bone metabolism, osteopathies, and fracture healing: challenges of complex models. Archives Toxicol. 94 (12), 3937–3958. 10.1007/s00204-020-02906-z PMC7655582 32910238

[B30] Elfawy L. A. Ng C. Y. Amirrah I. N. Mazlan Z. Wen A. P. Y. Fadilah N. I. M. (2023). Sustainable approach of functional biomaterials–tissue engineering for skin burn treatment: a comprehensive review. Pharmaceuticals 16, 701. 10.3390/ph16050701 37242483 PMC10223453

[B31] Feng G. Zha Z. Huang Y. Li J. Wang Y. Ke W. (2018). Sustained and bioresponsive two-stage delivery of therapeutic miRNA via polyplex micelle-loaded injectable hydrogels for inhibition of intervertebral disc fibrosis. Adv. Healthc. Mater 7, 1800623. 10.1002/adhm.201800623 30296017

[B32] Friedman R. C. Farh K. K. H. Burge C. B. Bartel D. P. (2009). Most mammalian mRNAs are conserved targets of microRNAs. Genome Res. 19, 92–105. 10.1101/gr.082701.108 18955434 PMC2612969

[B33] Frontera W. R. Ochala J. (2015). Skeletal muscle: a brief review of structure and function. Behav. Genet. 45, 183–195. 10.1007/s00223-014-9915-y 25294644

[B34] Gan M. Zhou Q. Ge J. Zhao J. Wang Y. Yan Q. (2021). Precise *in-situ* release of microRNA from an injectable hydrogel induces bone regeneration. Acta Biomater. 135, 289–303. 10.1016/j.actbio.2021.08.041 34474179

[B35] Gil C. J. Li L. Hwang B. Cadena M. Theus A. S. Finamore T. A. (2022). Tissue engineered drug delivery vehicles: methods to monitor and regulate the release behavior. J. Control. Release 349, 143–155. 10.1016/j.jconrel.2022.04.044 35508223

[B36] Goulet J. Richard-Denis A. Thompson C. Mac-Thiong J. M. (2019). Relationships between specific functional abilities and health-related quality of life in chronic traumatic spinal cord injury. Am. J. Phys. Med. Rehabil. 98, 14–19. 10.1097/phm.0000000000001006 30157080

[B37] Guan S. Zhang Z. Wu J. (2022). Non-coding RNA delivery for bone tissue engineering: progress, challenges, and potential solutions. iScience 25, 104807. 10.1016/j.isci.2022.104807 35992068 PMC9385673

[B38] Guelfi G. Capaccia C. Anipchenko P. Ciancabilla F. Oommen O. P. Bufalari A. (2024). Mimic miRNA and anti-miRNA activated scaffolds as a therapeutic strategy to promote bone, cartilage, and skin regeneration. Macromol. 2024 4, 165–189. 10.3390/macromol4020009

[B39] Hama R. Reinhardt J. W. Ulziibayar A. Watanabe T. Kelly J. Shinoka T. (2023). Recent tissue engineering approaches to mimicking the extracellular matrix structure for skin regeneration. Biomimetics 8, 130–138. 10.3390/biomimetics8010130 36975360 PMC10046023

[B40] Harris A. F. Lacombe J. Zenhausern F. Lukomska B. Walczak P. Oliveira J. M. (2021). The emerging role of decellularized plant-based scaffolds as a new biomaterial. Int. J. Mol. Sci. 22, 12347. 10.3390/ijms222212347 34830229 PMC8625747

[B41] Heinlen L. Humphrey M. B. (2017). Skeletal complications of rheumatoid arthritis. Osteoporos. Int. 28, 2801–2812. 10.1007/s00198-017-4170-5 28779302

[B42] Hoffman T. Khademhosseini A. Langer R. (2019). Chasing the paradigm: clinical translation of 25 Years of tissue engineering. Tissue Eng. Part A 25, 679–687. 10.1089/ten.tea.2019.0032 30727841 PMC6533781

[B43] Hosseinpour M. tanha G. K. Forouzanfar F. Moghbeli M. Enderami S. E. Saburi E. (2024). Application of natural polymers in skin tissue engineering using 3D scaffold. Int. J. Polym. Mater. Polym. Biomaterials, 1–12. 10.1080/00914037.2024.2335160

[B44] Hu H. Zhang H. Bu Z. Liu Z. Lv F. Pan M. (2022). Small extracellular vesicles released from bioglass/hydrogel scaffold promote vascularized bone regeneration by transferring miR-23a-3p. Int. J. Nanomedicine 17, 6201–6220. 10.2147/ijn.s389471 36531118 PMC9749034

[B45] Hu K. Xiang L. Chen J. Qu H. Wan Y. Xiang D. (2021). PLGA-liposome electrospun fiber delivery of miR-145 and PDGF-BB synergistically promoted wound healing. Chem. Eng. J. 422, 129951. 10.1016/j.cej.2021.129951

[B46] Huang H. Young W. Skaper S. Chen L. Moviglia G. Saberi H. (2020). Clinical neurorestorative therapeutic guidelines for spinal cord injury (IANR/CANR version 2019). J. Orthop. Transl. 20, 14–24. 10.1016/j.jot.2019.10.006 PMC6939117 31908929

[B47] Hutmacher D. W. Tandon B. Dalton P. D. (2023). Scaffold design and fabrication. Tissue Eng., 355–385. 10.1016/b978-0-12-824459-3.00011-1

[B48] Javan N. Ansari M. H. K. Dadashpour M. Khojastehfard M. Bastami M. Rahmati-Yamchi M. (2019). Synergistic antiproliferative effects of Co-nanoencapsulated curcumin and chrysin on MDA-MB-231 breast cancer cells through upregulating miR-132 and miR-502c. Nutr. Cancer 71, 1201–1213. 10.1080/01635581.2019.1599968 30955355

[B49] Jedari B. Rahmani A. Naderi M. Nadri S. (2020). MicroRNA-7 promotes neural differentiation of trabecular meshwork mesenchymal stem cell on nanofibrous scaffold. J. Cell Biochem. 121, 2818–2827. 10.1002/jcb.29513 31692062

[B50] Ke B. Eun S. Berkane Y. Olangian-Tehrani S. Mohammadyari F. Parvin S. (2023). Acellular dermal matrix in reconstructive surgery: applications, benefits, and cost. Front. Transplant. 2, 1133806. 10.3389/frtra.2023.1133806 38993878 PMC11235262

[B51] Kelly D. C. Raftery R. M. Curtin C. M. O’Driscoll C. M. O’Brien F. J. (2019). Scaffold-based delivery of nucleic acid therapeutics for enhanced bone and cartilage repair. J. Orthop. Research® 37, 1671–1680. 10.1002/jor.24321 31042304

[B52] Kim Y. S. Smoak M. M. Melchiorri A. J. Mikos A. G. (2019). An overview of the tissue engineering market in the United States from 2011 to 2018. Tissue Eng. Part A 25, 1–8. 10.1089/ten.tea.2018.0138 30027831 PMC6352506

[B53] Lacombe J. Harris A. F. Zenhausern R. Karsunsky S. Zenhausern F. (2020). Plant-based scaffolds modify cellular response to drug and radiation exposure compared to standard cell culture models. Front. Bioeng. Biotechnol. 8, 932. 10.3389/fbioe.2020.00932 32850759 PMC7426640

[B54] Lei L. Liu Z. Yuan P. Jin R. Wang X. Jiang T. (2019). Injectable colloidal hydrogel with mesoporous silica nanoparticles for sustained co-release of microRNA-222 and aspirin to achieve innervated bone regeneration in rat mandibular defects. J. Mater Chem. B 7, 2722–2735. 10.1039/c9tb00025a 32255005

[B55] Leng Q. Chen L. Lv Y. (2020). RNA-based scaffolds for bone regeneration: application and mechanisms of mRNA, miRNA and siRNA. Theranostics 10, 3190–3205. 10.7150/thno.42640 32194862 PMC7053199

[B56] Leng Q. Imtiyaz Z. Woodle M. C. Mixson A. J. (2023). Delivery of chemotherapy agents and nucleic acids with pH-dependent nanoparticles. Pharm. 2023 15, 1482. 10.3390/pharmaceutics15051482 PMC10222096 37242725

[B57] Lewis A. Koukoura A. Tsianos G. I. Gargavanis A. A. Nielsen A. A. Vassiliadis E. (2021). Organ donation in the US and Europe: the supply vs demand imbalance. Transpl. Rev. 35, 100585. 10.1016/j.trre.2020.100585 33071161

[B58] Li Q. Hu W. Huang Q. Yang J. Li B. Ma K. (2023). MiR146a-loaded engineered exosomes released from silk fibroin patch promote diabetic wound healing by targeting IRAK1. Signal Transduct. Target. Ther. 8 (1), 62–13. 10.1038/s41392-022-01263-w 36775818 PMC9922687

[B59] Li S. Qian T. Wang X. Liu J. Gu X. (2017). Noncoding RNAs and their potential therapeutic applications in tissue engineering. Engineering 3, 3–15. 10.1016/j.eng.2017.01.005

[B60] Li Y. Chen X. Jin R. Chen L. Dang M. Cao H. (2021). Injectable hydrogel with MSNs/microRNA-21-5p delivery enables both immunomodification and enhanced angiogenesis for myocardial infarction therapy in pigs. Sci. Adv. 7, 6740–6764. 10.1126/sciadv.abd6740 PMC7904259 33627421

[B61] Li Z. Meng Z. Zhao Z. (2022). Silk fibroin nanofibrous scaffolds incorporated with microRNA-222 loaded chitosan nanoparticles for enhanced neuronal differentiation of neural stem cells. Carbohydr. Polym. 277, 118791. 10.1016/j.carbpol.2021.118791 34893221

[B62] Limlawan P. Insin N. Marger L. Freudenreich M. Durual S. Vacharaksa A. (2023). 3D-printed TCP-HA scaffolds delivering MicroRNA-302a-3p improve bone regeneration in a mouse calvarial model. BDJ Open 2023 9 (1), 50–10. 10.1038/s41405-023-00177-1 PMC10673873 38001073

[B63] Liu X. Zhang L. Xu Z. Xiong X. Yu Y. Wu H. (2022). A functionalized collagen-I scaffold delivers microRNA 21-loaded exosomes for spinal cord injury repair. Acta Biomater. 154, 385–400. 10.1016/j.actbio.2022.10.027 36270583

[B64] Lolli A. Sivasubramaniyan K. Vainieri M. L. Oieni J. Kops N. Yayon A. (2019). Hydrogel-based delivery of antimiR-221 enhances cartilage regeneration by endogenous cells. J. Control. Release 309, 220–230. 10.1016/j.jconrel.2019.07.040 31369767

[B65] Madhusudanan P. Raju G. Shankarappa S. (2020). Hydrogel systems and their role in neural tissue engineering. J. R. Soc. Interface 17, 20190505. 10.1098/rsif.2019.0505 31910776 PMC7014813

[B66] Madry H. Venkatesan J. K. Carballo‐pedrares N. Rey‐rico A. Cucchiarini M. (2020). Scaffold-mediated gene delivery for osteochondral repair. Pharmaceutics 12, 930. 10.3390/pharmaceutics12100930 33003607 PMC7601511

[B67] Milbreta U. Lin J. Pinese C. Ong W. Chin J. S. Shirahama H. (2019). Scaffold-mediated sustained, non-viral delivery of miR-219/miR-338 promotes CNS remyelination. Mol. Ther. 27, 411–423. 10.1016/j.ymthe.2018.11.016 30611662 PMC6369635

[B68] Moncal K. K. Aydin R. S. T. Abu-Laban M. Heo D. N. Rizk E. Tucker S. M. (2019). Collagen-infilled 3D printed scaffolds loaded with miR-148b-transfected bone marrow stem cells improve calvarial bone regeneration in rats. Mater. Sci. Eng. C 105, 110128. 10.1016/j.msec.2019.110128 PMC6761997 31546389

[B69] Moraes F. C. Pichon C. Letourneur D. Chaubet F. (2021). miRNA delivery by nanosystems: state of the art and perspectives. Pharmaceutics 13, 1901. 10.3390/pharmaceutics13111901 34834316 PMC8619868

[B70] Mukund K. Subramaniam S. (2020). Skeletal muscle: a review of molecular structure and function, in health and disease. Wiley Interdiscip. Rev. Syst. Biol. Med. 12, e1462. 10.1002/wsbm.1462 31407867 PMC6916202

[B71] Mulholland E. J. McErlean E. M. Dunne N. McCarthy H. O. (2022). A Peptide/MicroRNA-31 nanomedicine within an electrospun biomaterial designed to regenerate wounds *in vivo* . Acta Biomater. 138, 285–300. 10.1016/j.actbio.2021.11.016 34800718

[B72] Muniyandi P. Palaninathan V. Mizuki T. Mohamed M. S. Hanajiri T. Maekawa T. (2021). Scaffold mediated delivery of dual miRNAs to transdifferentiate cardiac fibroblasts. Mater. Sci. Eng. C 128, 112323. 10.1016/j.msec.2021.112323 34474874

[B73] Muscolo A. Mariateresa O. Giulio T. Mariateresa R. (2024). Oxidative stress: the role of antioxidant phytochemicals in the prevention and treatment of diseases. Int. J. Mol. Sci. 25, 3264. 10.3390/ijms25063264 38542238 PMC10970659

[B74] Nazari B. Kazemi M. Kamyab A. Nazari B. Ebrahimi-Barough S. Hadjighassem M. (2020). Fibrin hydrogel as a scaffold for differentiation of induced pluripotent stem cells into oligodendrocytes. J. Biomed. Mater Res. B Appl. Biomater. 108, 192–200. 10.1002/jbm.b.34378 30957435

[B75] Negrini N. C. Toffoletto N. Farè S. Altomare L. (2020). Plant tissues as 3D natural scaffolds for adipose, bone and tendon tissue regeneration. Front. Bioeng. Biotechnol. 8, 550203. 10.3389/fbioe.2020.00723 PMC7344190 32714912

[B76] Nguyen L. H. Diao H. J. Chew S. Y. (2015). MicroRNAs and their potential therapeutic applications in neural tissue engineering. Adv. Drug Deliv. Rev. 88, 53–66. 10.1016/j.addr.2015.05.007 25980934

[B77] Oryan A. Monazzah S. Bigham-Sadegh A. (2015). Bone injury and fracture healing biology. Biomed. Environ. Sci. 28, 57–71. 10.3967/bes2015.006 25566863

[B78] Ouyang Z. Wei K. (2021). miRNA in cardiac development and regeneration. Cell Regen. 2021 10 (1), 14–21. 10.1186/s13619-021-00077-5 PMC8166991 34060005

[B79] Pan T. Song W. Xin H. Yu H. Wang H. Ma D. (2022). MicroRNA-activated hydrogel scaffold generated by 3D printing accelerates bone regeneration. Bioact. Mater 10, 1–14. 10.1016/j.bioactmat.2021.08.034 34901525 PMC8637000

[B80] Paternoster J. L. Vranckx J. J. (2022). State of the art of clinical applications of tissue engineering in 2021. Tissue Eng. Part B Rev. 28, 592–612. 10.1089/ten.teb.2021.0017 34082599

[B81] Peer N. Baatiema L. Kengne A. P. (2021). Ischaemic heart disease, stroke, and their cardiometabolic risk factors in Africa: current challenges and outlook for the future. Expert Rev. Cardiovasc Ther. 19, 129–140. 10.1080/14779072.2021.1855975 33305637

[B82] Pfeiffenberger M. Damerau A. Lang A. Buttgereit F. Hoff P. Gaber T. (2021). Fracture healing research—shift towards *in vitro* modeling? Biomedicines 9, 748. 10.3390/biomedicines9070748 34203470 PMC8301383

[B83] Płończak M. Wasyłeczko M. Jakutowicz T. Chwojnowski A. Czubak J. (2023). Intraarticular implantation of autologous chondrocytes placed on collagen or polyethersulfone scaffolds: an experimental study in rabbits. Polymers 15, 2360. 10.3390/polym15102360 37242936 PMC10221810

[B84] Pu M. Chen J. Tao Z. Miao L. Qi X. Wang Y. (2019). Regulatory network of miRNA on its target: coordination between transcriptional and post-transcriptional regulation of gene expression. Cell. Mol. Life Sci. 76, 441–451. 10.1007/s00018-018-2940-7 30374521 PMC11105547

[B85] Qi P. Niu Y. Wang B. (2021). MicroRNA-181a/b-1-encapsulated PEG/PLGA nanofibrous scaffold promotes osteogenesis of human mesenchymal stem cells. J. Cell Mol. Med. 25, 5744–5752. 10.1111/jcmm.16595 33991050 PMC8184675

[B86] Rahmani A. Naderi M. Barati G. Arefian E. Jedari B. Nadri S. (2020). The potency of hsa-miR-9-1 overexpression in photoreceptor differentiation of conjunctiva mesenchymal stem cells on a 3D nanofibrous scaffold. Biochem. Biophys. Res. Commun. 529, 526–532. 10.1016/j.bbrc.2020.06.006 32736669

[B87] Ribeiro A. Schoof C. Izzotti A. Pereira L. Vasques L. (2014). MicroRNAs: modulators of cell identity, and their applications in tissue engineering. Microrna 3, 45–53. 10.2174/2211536603666140522003539 25069512 PMC4262937

[B88] Rimann M. Bono E. Annaheim H. Bleisch M. Graf-Hausner U. (2016). Standardized 3D bioprinting of soft tissue models with human primary cells. J. Lab. Autom. 21, 496–509. 10.1177/2211068214567146 25609254

[B89] Roger V. L. (2021). Epidemiology of heart failure. Circ. Res. 128, 1421–1434. 10.1161/circresaha.121.318172 33983838

[B90] Roth G. A. Mensah G. A. Johnson C. O. Addolorato G. Ammirati E. Baddour L. M. (2020). Global burden of cardiovascular diseases and risk factors, 1990–2019: update from the GBD 2019 study. J. Am. Coll. Cardiol. 76, 2982–3021. 10.1016/j.jacc.2020.11.010 33309175 PMC7755038

[B91] Saleh B. Dhaliwal H. K. Portillo-Lara R. Sani E. S. Abdi R. Amiji M. M. (2019). Local immunomodulation using an adhesive hydrogel loaded with miRNA-laden nanoparticles promotes wound healing. Small 15, 1902232. 10.1002/smll.201902232 PMC6726510 31328877

[B92] Saliminejad K. Khorshid H. R. K. Fard S. S. Ghaffari S. H. (2019). An overview of microRNAs: biology, functions, therapeutics, and analysis methods. J. Cell Physiol. 234, 5451–5465. 10.1002/jcp.27486 30471116

[B93] Sener G. Hilton S. A. Osmond M. J. Zgheib C. Newsom J. P. Dewberry L. (2020). Injectable, self-healable zwitterionic cryogels with sustained microRNA - cerium oxide nanoparticle release promote accelerated wound healing. Acta Biomater. 101, 262–272. 10.1016/j.actbio.2019.11.014 31726250

[B94] Seyhan A. A. (2024). Trials and tribulations of MicroRNA therapeutics. Int. J. Mol. Sci. 25, 1469. 10.3390/ijms25031469 38338746 PMC10855871

[B95] Sharma V. Dash S. K. Govarthanan K. Gahtori R. Negi N. Barani M. (2021). Recent advances in cardiac tissue engineering for the management of myocardium infarction. Cells 10, 2538. 10.3390/cells10102538 34685518 PMC8533887

[B96] Shen K. Duan A. Cheng J. Yuan T. Zhou J. Song H. (2022). Exosomes derived from hypoxia preconditioned mesenchymal stem cells laden in a silk hydrogel promote cartilage regeneration via the miR-205–5p/PTEN/AKT pathway. Acta Biomater. 143, 173–188. 10.1016/j.actbio.2022.02.026 35202856

[B97] Silvestro S. Mazzon E. (2022). MiRNAs as promising translational strategies for neuronal repair and regeneration in spinal cord injury. Cells 11, 2177. 10.3390/cells11142177 35883621 PMC9318426

[B98] Song Y. Li M. Lei S. Hao L. Lv Q. Liu M. (2022). Silk sericin patches delivering miRNA-29-enriched extracellular vesicles-decorated myoblasts (SPEED) enhances regeneration and functional repair after severe skeletal muscle injury. Biomaterials 287, 121630. 10.1016/j.biomaterials.2022.121630 35816980

[B99] Stanco D. Urbán P. Tirendi S. Ciardelli G. Barrero J. (2020). 3D bioprinting for orthopaedic applications: current advances, challenges and regulatory considerations. Bioprinting 20, e00103. 10.1016/J.BPRINT.2020.E00103 PMC8609155 34853818

[B100] Šuca H. Čoma M. Tomšů J. Sabová J. Zajíček R. Brož A. (2024). Current approaches to wound repair in burns: how far have we come from cover to close? A narrative review. J. Surg. Res. 296, 383–403. 10.1016/j.jss.2023.12.043 38309220

[B101] Tang X. Sun C. (2020). The roles of MicroRNAs in neural regenerative medicine. Exp. Neurol. 332, 113394. 10.1016/j.expneurol.2020.113394 32628967

[B102] Tottoli E. M. Dorati R. Genta I. Chiesa E. Pisani S. Conti B. (2020). Skin wound healing process and new emerging technologies for skin wound care and regeneration. Pharmaceutics 12, 735. 10.3390/pharmaceutics12080735 32764269 PMC7463929

[B103] Uquillas J. A. Moroni L. de Boer J. (2023). An introduction to tissue engineering; the topic and the book. Tissue Eng., 1–12. 10.1016/b978-0-12-824459-3.00001-9

[B104] Van Der Ven C. F. T. Tibbitt M. W. Conde J. Van Mil A. Hjortnaes J. Doevendans P. A. (2021). Controlled delivery of gold nanoparticle-coupled miRNA therapeutics via an injectable self-healing hydrogel. Nanoscale 13, 20451–20461. 10.1039/d1nr04973a 34817483 PMC8675028

[B105] Veronesi F. Desando G. Fini M. Parrilli A. Lolli R. Maglio M. (2019). Bone marrow concentrate and expanded mesenchymal stromal cell surnatants as cell-free approaches for the treatment of osteochondral defects in a preclinical animal model. Int. Orthop. 43, 25–34. 10.1007/s00264-018-4202-6 30324310

[B106] Wan J. Liu H. Li J. Zeng Y. Ren H. Hu Y. (2023). PEG-SH-GNPs-SAPNS@miR-29a delivery system promotes neural regeneration and recovery of motor function after spinal cord injury. J. Biomater. Sci. Polym. Ed. 34, 2107–2123. 10.1080/09205063.2023.2230841 37366285

[B107] Wang H. Wan H. Wang Q. Ma Y. Su G. Cao X. (2023). Engineered multifunctional silk fibroin/gelatin hydrogel conduit loaded with miR-29a@ZIF-8 nanoparticles for peripheral nerve regeneration. Smart Mater Med. 4, 480–492. 10.1016/j.smaim.2023.02.002

[B108] Wang T. Y. Park C. Zhang H. Rahimpour S. Murphy K. R. Goodwin C. R. (2021). Management of acute traumatic spinal cord injury: a review of the literature. Front. Surg. 8, 698736. 10.3389/fsurg.2021.698736 34966774 PMC8710452

[B109] Watson E. C. Adams R. H. (2018). Biology of bone: the vasculature of the skeletal system. Cold Spring Harb. Perspect. Med. 8, a031559. 10.1101/cshperspect.a031559 28893838 PMC6027931

[B110] Wen M. Zhi D. Wang L. Cui C. Huang Z. Zhao Y. (2020). Local delivery of dual MicroRNAs in trilayered electrospun grafts for vascular regeneration. ACS Appl. Mater Interfaces 12, 6863–6875. 10.1021/acsami.9b19452 31958006

[B111] Wen M. Zhou F. Cui C. Zhao Y. Yuan X. (2019). Performance of TMC-g-PEG-VAPG/miRNA-145 complexes in electrospun membranes for target-regulating vascular SMCs. Colloids Surf. B Biointerfaces 182, 110369. 10.1016/j.colsurfb.2019.110369 31336282

[B112] Wu G. Sun B. Zhao C. Wang Z. Teng S. Yang M. (2021). Three-dimensional tendon scaffold loaded with *TGF-β1* gene silencing plasmid prevents tendon adhesion and promotes tendon repair. ACS Biomater. Sci. Eng. 7, 5739–5748. 10.1021/acsbiomaterials.1c00747 34723484

[B113] Wu H. Liao H. Li F. Lee J. Hu P. Shao W. (2020). Bioactive ROS-scavenging nanozymes for regenerative medicine: reestablishing the antioxidant firewall. Nano Sel. 1, 285–297. 10.1002/nano.202000021

[B114] Xiao D. Yang M. Zhang M. Rong L. Wang Y. Cheng H. (2021). MicroRNA-126 from stem cell extracellular vesicles encapsulated in a tri-layer hydrogel scaffold promotes bladder angiogenesis by activating CXCR4/SDF-1α pathway. Chem. Eng. J. 425, 131624. 10.1016/j.cej.2021.131624

[B115] Xu Y. Niu Y. Wu B. Cao X. Gong T. Zhang Z. R. (2021). Extended-release of therapeutic microRNA via a host-guest supramolecular hydrogel to locally alleviate renal interstitial fibrosis. Biomaterials 275, 120902. 10.1016/j.biomaterials.2021.120902 34087588

[B116] Yang L. Zhang L. Hu J. Wang W. Liu X. (2021). Promote anti-inflammatory and angiogenesis using a hyaluronic acid-based hydrogel with miRNA-laden nanoparticles for chronic diabetic wound treatment. Int. J. Biol. Macromol. 166, 166–178. 10.1016/j.ijbiomac.2020.10.129 33172616

[B117] Zeng J. H. Liu S. W. Xiong L. Qiu P. Ding L. H. Xiong S. L. (2018). Scaffolds for the repair of bone defects in clinical studies: a systematic review. J. Orthop. Surg. Res. 13, 33–14. 10.1186/s13018-018-0724-2 29433544 PMC5809923

[B118] Zhang N. Lin J. Lin V. P. H. Milbreta U. Chin J. S. Chew E. G. Y. (2021). A 3D fiber-hydrogel based non-viral gene delivery platform reveals that microRNAs promote axon regeneration and enhance functional recovery following spinal cord injury. Adv. Sci. 8, 2100805. 10.1002/advs.202100805 PMC8336488 34050637

[B119] Zhang N. Milbreta U. Chin J. S. Pinese C. Lin J. Shirahama H. (2019). Biomimicking fiber scaffold as an effective *in vitro* and *in vivo* MicroRNA screening platform for directing tissue regeneration. Adv. Sci. 6, 1800808. 10.1002/advs.201800808 PMC6498117 31065509

[B120] Zhao X. Song W. Chen Y. Liu S. Ren L. (2018). Collagen-based materials combined with microRNA for repairing cornea wounds and inhibiting scar formation. Biomater. Sci. 7, 51–62. 10.1039/c8bm01054d 30398231

[B121] Zhao Z. Liu D. Chen Y. Kong Q. Li D. Zhang Q. (2019). Ureter tissue engineering with vessel extracellular matrix and differentiated urine-derived stem cells. Acta Biomater. 88, 266–279. 10.1016/j.actbio.2019.01.072 30716556

[B122] Zhong R. Talebian S. Mendes B. B. Wallace G. Langer R. Conde J. (2023). Hydrogels for RNA delivery. Nat. Mater. 2023 22 (7), 818–831. 10.1038/s41563-023-01472-w PMC10330049 36941391

[B123] Zipser C. M. Cragg J. J. Guest J. D. Fehlings M. G. Jutzeler C. R. Anderson A. J. (2022). Cell-based and stem-cell-based treatments for spinal cord injury: evidence from clinical trials. Lancet Neurol. 21, 659–670. 10.1016/s1474-4422(21)00464-6 35569486

